# Targeting LINC02544/miR-497-5p/CAPRIN1 axis via exosome-based siRNA to overcome immunotherapy resistance in triple-negative breast cancer

**DOI:** 10.1186/s10020-025-01336-w

**Published:** 2025-08-16

**Authors:** Bin Lian, Jiayi Li, Shihui Tang, Ting Li, Jinping Li

**Affiliations:** 1 Department of Surgical Oncology, General Hospital of Ningxia Medical University, No. 804, Shengli South Str eet, Xingqing District, Yinchuan, 750004 China; 2 Northwest University for Nationalities, Lanzhou, 730030 China; 3 Department of Operating Room, General Hospital of Ningxia Medical Un iversity, Yinchuan, 750004 China; 4 Ningxia Medical University, Yinchuan, 750004 China

**Keywords:** Triple-negative breast cancer, LINC02544, MiR-497-5p, Immunotherapy resistance, Exosome-based SiRNA delivery

## Abstract

**Background:**

Triple-negative breast cancer (TNBC) is an aggressive breast cancer subtype characterized by the absence of estrogen receptor, progesterone receptor, and HER2 expression, leading to poor clinical outcomes and resistance to targeted therapies. Immunotherapy has shown limited success due to the development of resistance mechanisms. This study aimed to investigate the role of long non-coding RNA LINC02544 in mediating immunotherapy resistance through regulation of the miR-497-5p/CAPRIN1 axis in TNBC.

**Methods:**

Bioinformatic analyses of TCGA and GEO databases were performed to assess LINC02544 and miR-497-5p expression in TNBC tissues. In vitro experiments evaluated the regulatory effects of LINC02544 on miR-497-5p and CAPRIN1 expression, as well as TNBC cell proliferation and migration. Exosome-mediated siRNA delivery targeting LINC02544 (exo/si-LINC02544) was tested both in vitro and in vivo in combination with a PD-1 inhibitor in a TNBC mouse model.

**Results:**

LINC02544 was significantly overexpressed in TNBC, while miR-497-5p was downregulated. In vitro, LINC02544 silencing via exo/si-LINC02544 reduced CAPRIN1 levels, upregulated miR-497-5p, and inhibited TNBC cell proliferation and migration. In vivo, exo/si-LINC02544 combined with PD-1 blockade suppressed tumor growth and enhanced immune cell infiltration.

**Conclusions:**

Targeting the LINC02544/miR-497-5p/CAPRIN1 axis with exosome-based siRNA delivery represents a promising therapeutic strategy to overcome immunotherapy resistance in TNBC.

**Supplementary Information:**

The online version contains supplementary material available at 10.1186/s10020-025-01336-w.

## Highlights

1. This study is the first to report that LINC02544 regulates TNBC immune resistance through the miR-497-5p/CAPRIN1 axis.

2. This study finds that LINC02544 is significantly overexpressed in TNBC samples, exhibiting high diagnostic efficacy.

3. This study finds that miR-497-5p can target and regulate CAPRIN1, affecting TNBC cell behavior.

4. This study finds that exo/si-LINC02544 can reverse TNBC cell immune resistance.

5. This study provides new theoretical foundations and molecular targets for the diagnosis and treatment of TNBC.

**Supplementary Information:**

The online version contains supplementary material available at 10.1186/s10020-025-01336-w.

## Introduction

Triple-negative breast cancer (TNBC) is a highly aggressive subtype of breast cancer characterized by the lack of estrogen receptors (ER), progesterone receptors (PR), and human epidermal growth factor receptor 2 (HER2). Consequently, traditional endocrine and targeted therapies are ineffective against TNBC (Derakhshan and Reis-Filho 2022; Hong and Xu 2022; Zhu et al. 2023). TNBC accounts for approximately 10–20% of all breast cancer cases and has a higher incidence among younger women, African American women, and BRCA1 mutation carriers (Ferrari et al. 2022; Martelli et al. 2023; Shi et al. 2023). Due to the absence of specific biomarkers, treatment primarily relies on surgery and chemotherapy (Leon-Ferre and Goetz 2023; Xiao et al. 2022; Nelson et al. 2022). However, TNBC exhibits heterogeneous responses to chemotherapy, often accompanied by severe side effects and a high recurrence rate (Burguin et al. 2021; Fan et al. 2024; Zhang et al. 2021). Thus, developing new therapeutic strategies and targeted drugs is crucial for improving the prognosis of TNBC patients (Yang et al. 2023; Li et al. 2022; Howard and Olopade 2021).

In recent years, immunotherapy has emerged as a cutting-edge approach in cancer treatment, aiming to activate the patient’s immune system to recognize and attack cancer cells (Liu et al. 2024). However, the response rate of TNBC patients to immune checkpoint inhibitors, such as PD-1/PD-L1 inhibitors, remains low. This may be attributed to the complexity of the TNBC tumor microenvironment, immunosuppressive conditions, and tumor cell heterogeneity (Zhu et al. 2021; Salas-Benito et al. 2021; Debien et al. 2023). Studies have shown that TNBC harbors a significant number of immunosuppressive cells, such as regulatory T cells (Tregs) and myeloid-derived suppressor cells (MDSCs), which inhibit T cell function through various mechanisms, leading to immunotherapy resistance (Liu et al. 2023a, b; Fu et al. 2022; Baldominos et al. 2022). Additionally, the lack of sufficient tumor-infiltrating lymphocytes (TILs) in the TNBC tumor microenvironment contributes to poor immunotherapy response (van den Ende et al. 2023; Geurts and Kok 2023; Baldominos et al. 2022). Therefore, understanding and overcoming the mechanisms of immunotherapy resistance in TNBC is vital for enhancing treatment efficacy (Zheng et al. 2023).

Long non-coding RNAs (lncRNAs) play crucial roles in gene expression regulation, cell differentiation, and disease development (de Goede et al. 2021; Wang et al. 2024; Mikaeili et al. 2023). LINC02544, an emerging lncRNA, has been found to be abnormally expressed in various types of cancer (Wei et al. 2022; Fan et al. 2022). However, its specific functions and regulatory mechanisms in TNBC remain unclear (Wei et al. 2022; Fan et al. 2022). Previous studies suggest that LINC02544 may influence gene expression and cellular behavior through interactions with microRNAs (miRNAs). Notably, LINC02544 has been shown to regulate processes such as cell proliferation, migration, and invasion in certain cancers (Wei et al. 2022; Fan et al. 2022). Therefore, investigating the role and regulatory network of LINC02544 in TNBC is essential for understanding its function in cancer.

This study employs advanced technologies to comprehensively elucidate the mechanisms of LINC02544 in TNBC. Initially, we downloaded the Breast Invasive Carcinoma (BRCA) dataset from the The Cancer Genome Atlas (TCGA) database, selecting 116 TNBC samples and 113 normal breast tissue samples to form the TCGA-TNBC dataset. We conducted statistical analyses on the lncRNA expression profiles of these samples to identify differentially expressed genes (DEGs) and used Least Absolute Shrinkage and Selection Operator (LASSO) regression for variable selection. Subsequently, we quantified the infiltration of various immune cell types in the TNBC dataset. Using the LncBook database, we annotated potential miRNA targets of LINC02544 and identified key miRNAs by integrating differentially expressed miRNA data from the TCGA-TNBC dataset. Additionally, we downloaded the TNBC single-cell sequencing (scRNA-seq) dataset from the Gene Expression Omnibus (GEO) database, annotated malignant epithelial cells, and identified key mRNA targets of miRNAs using the TCGA-TNBC dataset. In the experimental phase, we cultured TNBC cell lines and conducted dual-luciferase reporter assays to verify the direct regulatory effect of LINC02544 on miR-497-5p and the targeting of CAPRIN1 by miR-497-5p. We extracted exosomes (eoxs) secreted by TNBC cell lines using ultracentrifugation and characterized their size and morphology through nanoparticle tracking analysis (NTA), transmission electron microscopy (TEM), and Western Blot (WB). Fluorescently labeled LINC02544 siRNA (si-LINCO2544) was loaded into exos via electroporation, and uptake efficiency was assessed using fluorescence microscopy and flow cytometry. Cancer cell-derived exosomes have become preferred vehicles for siRNA delivery due to their enhanced permeability, biocompatibility, prolonged circulation half-life, and reduced toxicity and immunogenicity. These properties facilitate targeted therapy and effective gene knockdown in cancer management, thereby improving therapeutic outcomes (Ubanako et al. 2024).

This study aims to elucidate the mechanisms by which LINC02544 contributes to immunotherapy resistance in TNBC through the miR-497-5p/CAPRIN1 axis and to explore the potential of exosome-loaded LINC02544 siRNA (exo/si-LINC02544) in reversing this resistance. Through systematic analysis and experimental validation, this research not only identifies new potential targets for TNBC treatment but also proposes innovative approaches for the application of exos in cancer therapy. Experimental results demonstrate that exo/si-LINC02544 significantly reduce the expression of LINC02544 and CAPRIN1 in resistant TNBC cells, increase miR-497-5p expression, and significantly inhibit cell proliferation and migration. Additionally, combined treatment with exo/si-LINC02544 and PD-1 inhibitors markedly suppressed tumor growth and enhanced immune cell infiltration in mice. These findings indicate that exo/si-LINC02544 activate immune cell infiltration, thereby reducing immune resistance and ultimately inhibiting the growth of resistant TNBC tumors. This study provides new potential targets and therapeutic strategies for treating TNBC immune resistance and offers important scientific and clinical insights into the application of exos in cancer therapy.

## Materials and methods

### Public data download

Data was downloaded from the TCGA database (https://cancergenome.nih.gov/), specifically the TCGA-BRCA (The Cancer Genome Atlas, Breast Invasive Carcinoma) expression profiles and clinical information. We identified TNBC samples by filtering for cases where phenotype_file$breast_carcinoma_estrogen_receptor_status (estrogen receptor, ER), phenotype_file$breast_carcinoma_progesterone_receptor_status (progesterone receptor, PR), and phenotype_file$lab_proc_her2_neu_immunohistochemistry (IHC)_receptor_status (human epidermal growth factor receptor, HER2) were all “Negative,” resulting in a total of 116 TNBC samples.

Using the “TCGAbiolinks” package in R, we obtained mRNA-seq, lncRNA-seq, and miRNA-seq expression profiles. The mRNA and lncRNA expression profiles included 115 TNBC samples and 113 normal samples, while the miRNA expression profile contained 113 TNBC samples and 104 normal samples.

Additionally, TNBC-related scRNA-seq data was retrieved from the GEO (https://www.ncbi.nlm.nih.gov/geo/) under the dataset GSE221743, selecting one TNBC sample (GSM6893838).

Since these data were obtained from public databases, no ethical approval or informed consent was required.

### Differential analysis

We performed differential gene expression analysis between the TNBC and Normal groups using the LIMMA (Linear Models for Microarray Data) package in R software. Genes with|logFC|>1 and *p*.adjust < 0.05 were considered significantly differentially expressed. Visualization of the DEGs was carried out through volcano plots created using the “ggplot2” package. All analyses were conducted using R version 4.3.1 (R Foundation for Statistical Computing).

### Least absolute shrinkage and selection operator (LASSO) regression

LASSO regression is a method used in regression models for variable selection and regularization. It involves selecting the optimal regularization parameter (λ) through cross-validation by fitting the model on a range of λ values and choosing the best performing one. This approach examines the coefficients under the selected λ value. LASSO regression tends to shrink some coefficients to zero, aiding in variable selection. Perform LASSO regression analysis using the “glmnet” package in R software.

### Analysis of receiver operating characteristic (ROC) curves and survival analysis

ROC curve analysis and survival analysis were conducted using the training and validation datasets. The ROC curves were generated using the pROC package in R programming language, based on the expression values of candidate genes. This evaluation aimed to assess the accuracy of predicting the disease status of samples based on gene expression levels.

### Single-sample gene set enrichment analysis (ssGSEA)

To comprehensively investigate the overall immune status of TCGA-TNBC, we employed the ssGSEA algorithm to assess immune cell infiltration and immune function. The ssGSEA method was utilized to evaluate the abundance of 28 immune cell infiltrates in TCGA-TNBC, with grouping based on the expression levels of LINC02544 to illustrate the immune cell infiltration status between different groups.

### Analysis of the ceRNA network of incRNA-miRNA-mRNA

The LncBook dataset was utilized to investigate the miRNA targets of LINC02544, leading to the annotation of 181 target miRNAs. A comparative analysis was conducted by intersecting these with significantly downregulated miRNAs in TCGA-TNBC. The VennDiagram package version 1.6.20 in the R environment was employed to visually represent the intersection of the two groups.

Using the miRDB database (http://mirdb.org/) and the TargetScan database (https://www.targetscan.org/vert_80/), the mRNA targets of miR-497-5p were predicted. The miRDB database annotated 1357 mRNA target genes, while the TargetScan database annotated 207 mRNA target genes.

### Cluster analysis

To reduce the dimensionality of the scRNA-Seq dataset, a Principal Component Analysis (PCA) was performed based on the variance of the top 2000 highly variable genes. The top 20 principal components (PCs) were selected for downstream analysis using the Elbowplot function in the Seurat package. The Seurat package’s FindClusters function was applied to identify major cell subpopulations with a resolution set to the default value (res = 1). Subsequently, the scRNA-seq sequencing data were non-linearly dimensionally reduced using the UMAP algorithm. Markers for various cell subpopulations were selected using the Seurat package. Cell annotation was conducted using the CellMarker online resource in conjunction with the “SingleR” package, considering known cell lineage-specific marker genes. Finally, intercellular communication analysis was performed using the “CellChat” package in R.

The R package “inferCNV” was utilized to assess copy number variations (CNVs) in individual cells. This tool aims to infer the instability of copy numbers by analyzing tumor scRNA-seq data. In essence, inferCNV compares gene expression levels in malignant cells to those in normal cells, using cell subpopulations other than epithelial cells as references while excluding sex chromosomes.

The FindAllMarkers function was employed to analyze feature genes in each cell type post-clustering, focusing on selecting feature genes for malignant epithelial cells.

### Weighted gene co-expression network analysis (WGCNA)

Initially, we utilized gene expression profiles (based on feature genes of malignant cells from a scRNA-seq dataset) to compute the Median Absolute Deviation (MAD) for each gene. Subsequently, we excluded the bottom 50% of genes with the smallest MAD values. Outliers in genes and samples were then removed using the goodSamplesGenes function from the R software package WGCNA. Next, a scale-free co-expression network was constructed with WGCNA, setting a minimum gene dendrogram height of 30 and a sensitivity of 3. Additionally, modules with a distance less than 0.25 were merged, resulting in the identification of 5 co-expression modules, with the grey module considered as a gene set that could not be assigned to any module.

Subsequently, the correlation between modules and groups was analyzed using Pearson correlation testing (*p* < 0.05). Among them, genes in the most significant module were identified as TNBC-associated genes for further analysis.

### Analysis of the correlation between CAPRIN1 and immune checkpoint genes in pan-cancer

We obtained a uniformly standardized pan-cancer dataset from the UCSC database (https://xenabrowser.net/), which included data from TCGA, TARGET, and GTEx (pan-cancer data, *n* = 19,131, number of genes = 60,499). From this dataset, we extracted the expression data of ENSG00000135387 (CAPRIN1) gene and 60 immune checkpoint pathway genes (24 Inhibitory and 36 Stimulatory) in each sample. Samples originating from Primary Solid Tumor, Primary Tumor, Primary Blood Derived Cancer-Bone Marrow, and Primary Blood Derived Cancer-Peripheral Blood were selected. We filtered out all normal samples and applied a log2(x + 0.001) transformation to each expression value. Subsequently, we calculated the Pearson correlation between ENSG00000135387 (CAPRIN1) and marker genes of five immune pathways. The results were visualized using ggplot2, where * denotes a *p*-value < 0.05.

### Cell culturing

The MDA-MB-231 (CRM-HTB-26) human breast cancer cell line was obtained from the American Type Culture Collection (ATCC). The cells were cultured in RPMI-1640 medium (11875093, Gibco, Thermo Fisher Scientific) supplemented with 10% fetal bovine serum (A5670701, Gibco, Thermo Fisher Scientific), 100 U/mL penicillin, and 100 U/mL streptomycin (15070063, Gibco, Thermo Fisher Scientific), and maintained in a humidified cell culture incubator at 37 °C with 5% CO_2_. Upon reaching 80% confluence, cells were passaged using 0.25% trypsin/EDTA (25200056, Gibco, Thermo Fisher Scientific) for digestion.

The generation of the anti-PD-1 cell line proceeded as follows: MDA-MB-231 cancer cells (0.5 × 10^6^ cells in 50 µL sterile phosphate-buffered saline) were subcutaneously injected into the legs of huHSC-NOG-EXL mice (15–25 g) purchased from Beijing Vital River Laboratory Animal Technology Co., Ltd. (Beijing, China). Subsequently, the mice were intraperitoneally injected with Pembrolizumab: a dosage of Pembrolizumab (10 mg/kg) (Tamoxifen purchased from MedChemExpress, catalog number HY-P9902A) (Liu et al. 2021a, b), starting from day 4 post-tumor cell inoculation and administered twice a week for a total of 4 or 5 injections. A non-responsive tumor was isolated from mice treated with Pembrolizumab. The non-responsive tumor was dissociated into single cells, cultured in vitro for approximately 2 to 3 weeks, then re-injected into mice for 4 cycles of consecutive in vivo passaging with continuous Pembrolizumab treatment. These cells exhibited resistance to Pembrolizumab treatment in vivo and were designated as the MDA-MB-231/PEM cell line (Wang et al. 2017).

Human embryonic kidney (HEK293) cells (CL-0005, Wuhan Procell Life Science & Technology Co., Ltd.) were maintained in high-glucose Dulbecco’s Modified Eagle Medium (DMEM, WELGENE) supplemented with 10% fetal bovine serum (FBS) and penicillin-streptomycin. Cells were cultured at 37 °C in a humidified atmosphere containing 5% CO₂ (Arab et al. 2024).

### Preparation of exo/si-LINC02544

Exos were isolated from the conditioned media of the TNBC cell line, MDA-MB-231, using differential centrifugation method. The process involved incubating MDA-MB-231 cells in exo-free media for 48 h. Subsequently, the cell culture supernatant was collected and centrifuged at 800 × g for 5 min to remove dead cells, followed by centrifugation at 1500 × g for 15 min to eliminate cell debris, and then at 15,000 × g for 30 min to remove larger cell-derived vesicles. The resulting supernatant was ultra-centrifuged at 150,000 × g for 2 h to isolate exos. The morphology of purified exos was observed using TEM (Tecnai T10, FEI, Blackwood, NJ). Protein levels of exo surface markers were evaluated using antibodies against CD9 (ab263019, 1:1000, rabbit, Abcam), CD63 (ab134045, 1:1000, rabbit, Abcam), and TSG101 (ab125011, 1:2000, rabbit, Abcam) through WB analysis. Furthermore, the size distribution of exos was examined using NTA and NanoSight LM10 instrument (Malvern Panalytical, Malvern, UK).

For the knockdown of LINC02544 (si-LINC02544), siRNA oligonucleotides labeled with FAM were designed and synthesized by GenePharma (Shanghai, China).

si-LINC02544-1:SS 5’-GGAGGUGCUUCAAGUUGAAGG-3’;AS:5’-UUCAACUUGAAGCACCUCCUG-3’.

si-LINC02544-1:SS 5’-GGUUUGUUACAUAUGUAUACA-3’;AS:5’-UAUACAUAUGUAACAAACCUG-3’.

si-LINC02544-1:SS 5’-GGUGCACAGAAGUCAAGAACU-3’;AS:5’-UUCUUGACUUCUGUGCACCUG-3’.

The Gene Pulser X cell electroporation system (165–2660, Bio-Rad) was utilized to load si-LINC02544 into exos via electroporation. Exos with a total protein concentration of 20 µg were mixed with 20 µg of si-LINC02544 in 400 µL of PBS (pH 7.3), electroporated at 400 V, and immediately transferred to ice. Exos were ultra-centrifuged at 100,000 × g for 1 h to remove unloaded si-LINC02544. The precipitated exos, now loaded with si-LINC02544, were resuspended, termed as Exo/si-LINC02544. Subsequently, Exo/si-LINC02544 was obtained following the same procedure.

### Characterization of exo/si-LINC02544

Exo loading efficiency of FAM-labeled si-LINC02544 was quantified by electroporating it into exos followed by fluorescence measurement using a Molecular Devices fluorometer (Sunnyvale, CA). The calculation formula for loading efficiency is as follows: Loading Efficiency (%) = (Amount of si-LINC02544 post-electroporation)/(Total amount of si-LINC02544 pre-electroporation) × 100%.

In assessing the stability of siRNA, exo/si-LINC02544 was co-incubated with 0.01 µg/mL RNase A (ST578, Beyotime) at 37 °C for 45 min, then heparin (HY-17,567, MCE; 10 µL, 10 mg/mL) was added to release siRNA (based on heparin mimicking the polyionic structure of nucleic acids to interact with proteins, thereby separating siRNA from exos). The degradation products of siRNA were analyzed by polyacrylamide gel electrophoresis and detected using Packard Instant Image.

Furthermore, the transfection efficiency of si-LINC02544 on exos was examined. Following the aforementioned procedure, FAM-si-LINC02544 was loaded into exos. Exo/si-LINC02544 was added to cells and cultured at 37 °C for 4 h. The medium was refreshed, and cells were further cultured for 20 h, followed by flow cytometry analysis of FAM-positive cells for fluorescence intensity.

Dil dye (C1036, Beyotime, 5 µM) was added to Exo/si-LINC02544 to assess exo uptake. The precipitate obtained after repeated centrifugation was Dil-labeled exos. When human MDA-MB-231 cells reached 50% confluency, they were co-incubated with Dil-labeled Exo/si-LINC02544 at 37 °C for 24 h. Subsequently, fluorescence intensity was examined using a fluorescence microscope after staining with 4’,6-diamidino-2-phenylindole (DAPI, 2 µg/mL, C1005, Beyotime).

### Cell transfection and grouping

Upon reaching 60–70% confluence, MDA-MB-231/PEM cells were transfected with si-LINC02544 or siRNA negative control (si-RNA) using Lipofectamine 3000 reagent (L3000015, Invitrogen, USA). Transfection was carried out following the manufacturer’s instructions. Cells were collected 48 h post-transfection for subsequent analysis.

The commercialized CAPRIN1 shRNA lentivirus particles (sc-72785-V) and control lentivirus particles sh-NC were purchased from Santa Cruz Biotechnology (Shanghai) and titrated to 10^9^ TU/mL. MDA-MB-231 cells were seeded at 1 × 10^6^ cells per well in 6-well plates and incubated for 24 h. Following incubation, the cells were infected with the lentivirus. After 72 h post-infection, the medium was replaced with fresh medium containing 4 µg/mL puromycin (Invitrogen, A1113803), and the MDA-MB-231 cells were cultured for at least 14 days. Puromycin-resistant cells were then expanded in medium containing 2 µg/mL puromycin (Invitrogen, A1113803) for 9 days before being transferred to puromycin-free medium. This process yielded stable CAPRIN1 knockdown MDA-MB-231 cells. The efficiency of CAPRIN1 knockdown was verified by WB analysis.

miR-497-5p inhibitor (4464084), miR-497-5p mimic (4464066), mimic NC, and inhibitor NC were purchased from Thermo Fisher Scientific. These constructs were generated into lentivirus vectors carrying EGFP/Puromycin by Shanghai Hanheng Biotechnology Engineering Co., Ltd. MDA-MB-231 cells were seeded at 1 × 10^6^ per well in a 6-well plate, incubated for 24 h, then infected with lentivirus. Following viral infection for 72 h, the medium was replaced with medium containing 4 µg/mL puromycin (Invitrogen, A1113803), and cells were cultured for at least 14 days. Puromycin-resistant cells were amplified for 9 days in puromycin-supplemented medium, then transferred to puromycin-free medium to establish stable knockdown or overexpression of miR-497-5p in MDA-MB-231 cells. The knockdown and overexpression efficiency of miR-497-5p were verified using qPCR.

The cells were classified into the following groups based on the treatments: (1) si-LINC02544 group (MDA-MB-231/PEM cells treated with si-LINC02544); (2) si-NC group (MDA-MB-231/PEM cells treated with si-NC); (3) miR-497-5p mimic group (MDA-MB-231/PEM cells treated with miR-497-5p mimic); (4) mimic NC group (MDA-MB-231/PEM cells treated with mimic NC); (5) inhibitor NC group (MDA-MB-231/PEM cells treated with inhibitor NC); (6) miR-497-5p inhibitor group (MDA-MB-231/PEM cells treated with miR-497-5p inhibitor); (7) exo group (MDA-MB-231/PEM cells treated with exo); (8) exo/si-LINC02544 group (MDA-MB-231/PEM cells treated with exo/si-LINC02544); (9) exo + inhibitor NC + sh-NC group (MDA-MB-231/PEM cells treated with exo and infected with inhibitor NC and sh-NC lentivirus); (10) exo/si-LINC02544 + inhibitor NC + sh-NC group (MDA-MB-231/PEM cells treated with exo/si-LINC02544 and infected with inhibitor NC and sh-NC lentivirus); 11) exo/si-LINC02544 + miR-497-5p inhibitor + sh-NC group (MDA-MB-231/PEM cells treated with exo/si-LINC02544 and infected with miR-497-5p inhibitor and sh-NC lentivirus); 12) exo/si-LINC02544 + miR-497-5p inhibitor + sh-CAPRIN1 group (MDA-MB-231/PEM cells treated with exo/si-LINC02544 and infected with miR-497-5p inhibitor and sh-CAPRIN1 lentivirus).

### Quantitative reverse transcription polymerase chain reaction (qRT-PCR)

RNA was extracted from cells and tumor tissues using Trizol reagent (catalog no. 15596026, Invitrogen, Thermo Fisher Scientific, USA), and the concentration and purity of the extracted RNA were determined using a Nanodrop 2000 spectrophotometer (model 1011U, Nanodrop, USA). The RNA was reverse transcribed into cDNA following the instructions of the PrimeScript RT reagent Kit (catalog no. RR047A, Takara, Japan) under the conditions: 37 °C for 30–50 min followed by 85 °C for 5 s. Subsequently, RT-qPCR analysis was performed using the Fast SYBR Green PCR Kit (catalog no. RR820A, Takara, Japan) and the ABI PRISM 7300 RT-PCR system (Applied Biosystems, Thermo Fisher Scientific, USA). The PCR conditions included an initial denaturation at 95 °C for 5 min, followed by 40 cycles of denaturation at 95 °C for 30 s, annealing at 57 °C for 30 s, and extension at 72 °C for 30 s. Each reaction was set up in triplicate with LINC02544 and CAPRIN1 genes normalized against GAPDH, and miR-497-5p against U6. The relative gene expression levels were analyzed using the 2^-ΔΔCt^ method, where ΔΔCt = (average Ct value of the target gene in the experimental group - average Ct value of the reference gene in the experimental group) - (average Ct value of the target gene in the control group - average Ct value of the reference gene in the control group). The experiment was repeated three times. The primers were designed and synthesized by Shanghai Shenggong Bioengineering Co., Ltd as listed in Table S1.

### WB

Cells and tissues from different groups were collected and lysed in RIPA buffer containing 1% PMSF (P0013B, Beyotime, Shanghai, China) on ice for 30 min, followed by centrifugation at 14,000 ×g at 4 °C to collect the supernatant. The protein concentration in the samples was determined using the BCA method (P0012S, Beyotime, Shanghai, China). Subsequently, an appropriate amount of 5× loading buffer was added, and the proteins were denatured by boiling at 100 °C for 10 min, with a protein loading amount of 50 µg. Electrophoresis was conducted using a separating gel and a concentrating gel, and after electrophoresis, the protein bands of interest were transferred to a PVDF membrane. The PVDF membrane was then incubated with 5% skim milk powder at room temperature for 1 h, followed by overnight incubation at 4 °C with primary antibodies rabbit anti-CAPRIN1 (ab244360, Abcam, USA), anti-PD-L1 (ab205921, Abcam, USA), anti-CTLA-4 (ab237712, Abcam, USA), anti-Calnexin(ab22595, Abcam, USA), and anti-β-Tublin(ab6046, Abcam, USA). β-Tublin served as an internal control. After washing with PBST at room temperature, the membrane was incubated with an HRP-conjugated goat anti-rabbit IgG secondary antibody (ab205718, Abcam, UK). Signal detection was performed using an ECL detection system (32209, Thermo Fisher Scientific, USA) and exposure in an Amersham Imager 600 (USA). Subsequently, grayscale analysis was carried out using Image J. The experiment was repeated three times.

### Fluorescent reporter gene assay

Initially, Shanghai GenePharma constructed the LINC02544 WT, LINC02544 MT, CAPRIN1 3’UTR WT, or CAPRIN1 3’UTR MT into the report gene vector PGL3-basic. The reporter plasmid was then transfected into HEK293T cells (CL-0005, Wuhan PuNuoSi) along with miR-497-5p mimic or control for co-transfection. Additionally, each sample was co-transfected with the TK-Renilla reporter plasmid (Promega) as an internal control. After 48 h of transfection, the cells were washed twice with PBS and then their activities of firefly and Renilla luciferase were measured using the Synergy Neo2 HTS multimode microplate reader (Biotek, Winooski, VT, USA) following the instructions of the Dual-Luciferase Reporter Assay System (E1910, Promega).

### Cell viability assay using cell counting kit-8 (CCK-8)

The cell viability was assessed using the CCK-8 (Beyotime, C0037, Shanghai, China). Cells were seeded in a 96-well plate. After 24, 48, 72, and 96 h of treatment, 10 µL of CCK-8 solution was added to each well. The cells were then further incubated for 1–2 h. The absorbance of cells at a wavelength of 450 nm was measured. Each group consisted of 6 replicates, and the experiment was repeated thrice.

### Scratch assay experiment

Cells were seeded in a 6-well plate at a density of 8 × 10^5^ cells per well. After creating scratches using a 200 µL pipette tip, the cells were cultured in RPMI-1640 medium containing 5% FBS for 24 h. Scratch images of each well at 0 and 24 h were captured using an inverted microscope (Eclipse Ti2-E, Nikon). Each experiment was repeated three times. The width of each scratch was measured using ImageJ software, and the healing rate was calculated as follows: Healing Rate = ((Width of scratch at 0 h - Width of scratch at 24 h)/Width of scratch at 0 h) × 100%. The experiment was repeated three times.

### Transwell invasion assay

The Transwell invasion assay was conducted using Transwell chambers (8 μm pore size; Corning, USA) for in vitro cell invasion assessment. Matrigel-coated 8 μm pore size polycarbonate membrane Transwell chambers were prepared by adding 600 µL of FBS-containing medium to the lower chamber and equilibrating at 37 ℃ for 1 h. Digestion-passaged cells were resuspended in FBS-free DMEM medium, and 2 × 10^4^ cells/mL were seeded into the upper chamber. The chambers were then incubated at 37 ℃ with 5% CO_2_ for 24 h. Subsequently, the Transwell chambers were removed, washed twice with PBS, fixed with 5% glutaraldehyde at 4 ℃, stained with 0.1% crystal violet for 5 min, rinsed with PBS, surface cells were wiped off with a cotton ball, and the chambers were inverted for observation under a fluorescence microscope (Nikon TE2000, China). Five random fields were chosen for photography, and the average number of cells crossing the chamber was calculated per group. Each experiment was performed in triplicate.

### Flow cytometry analysis of cell cycle and apoptosis

Cell Cycle: Cells in suspension were centrifuged at 200 g for 3 min and washed twice with PBS, followed by fixation overnight at 4 °C in 70% ice-cold ethanol. Subsequently, cells were stained with propidium iodide (PI) using a cell cycle detection kit (BD Biosciences, Franklin Lakes, NJ, USA) and analyzed using a BD AccuriC6 flow cytometer (BD Biosciences). Finally, cell cycle distribution was analyzed using Modfit software (Verity Software House, Topsham, ME, USA).

Apoptosis: Apoptotic cells were analyzed using the Annexin V-FITC apoptosis detection kit (C1062S, Beyotime, Shanghai, China) following the manufacturer’s instructions. Cells were washed with PBS, centrifuged at 200 g for 3 min, supernatant was discarded, and cells were resuspended in 200 µL binding buffer containing 5 µL Annexin V and 10 µL PI. Subsequently, apoptotic cell rates were measured using a BD AccuriC6 flow cytometer (BD Biosciences).

### Construction of immunotherapy-resistant TNBC xenograft model

Forty huHSC-NOG-EXL mice (15–25 g) were purchased from Beijing Vitalstar Biotechnology Co., Ltd. (Beijing, China) and housed in SPF-grade animal facilities with a humidity of 60–65% and a temperature range of 22 to 25 °C. The mice were acclimated for one week before the experiment, and their health status was monitored. The experimental procedures and animal usage protocols were approved by the relevant ethics committee.

In the mammary fat pad of the mice, we implanted a suspension of 1 × 10^6^ MDA-MB-231/PEM cells that had been successfully transfected with miR-497-5p inhibitor, sh-CAPRIN1, and empty vector to establish an immunotherapy-resistant TNBC xenograft model. All xenograft mice received PEM treatment. The mice were divided into the following groups: exo + inhibitor NC + sh-NC group (injection of MDA-MB-231/PEM cells transfected with empty vector, receiving Pembrolizumab and exo blank treatment, *n* = 6), exo/si-LINC02544 + inhibitor NC + sh-NC group (injection of MDA-MB-231/PEM cells transfected with empty vector, receiving Pembrolizumab and exo/si-LINC02544 treatment, *n* = 6), exo/si-LINC02544 + miR-497-5p inhibitor + sh-NC group (injection of MDA-MB-231/PEM cells transfected with miR-497-5p inhibitor and sh-NC, receiving Pembrolizumab and exo/si-LINC02544 treatment, *n* = 6), exo/si-LINC02544 + miR-497-5p inhibitor + sh-CAPRIN1 group (injection of MDA-MB-231/PEM cells transfected with miR-497-5p inhibitor and sh-CAPRIN1, receiving Pembrolizumab and exo/si-LINC02544 treatment, *n* = 6). Tumor growth was monitored weekly by measuring the width (W) and length (L) of the tumors using a caliper, and tumor volume (V) was calculated using the formula V =(W^2^ × L)/2. Mice were euthanized when the tumor diameter exceeded 20 mm, and tumor tissues were used for subsequent experimental analysis (Li et al. 2020).

Drug treatment methods were as follows: when the tumor volume reached 20 mm^3^, Pembrolizumab or exo treatment was administered. Pembrolizumab treatment involved biweekly intraperitoneal injections for two weeks at a dose of Pembrolizumab (10 mg/kg) each time (Tamoxifen purchased from MedChemExpress, catalog number HY-P9902A) (Liu et al. 2021a, b). For exo treatment, 15 µg was administered via tail vein injection twice a week for two weeks (Wang et al. 2022).

### IHC

Tumor tissues were fixed in a 4% formaldehyde solution (F1635, Sigma-Aldrich) overnight, dehydrated, cleared, embedded in paraffin, and cut into 5 μm sections. The sections were deparaffinized in xylene and rehydrated in graded ethanol. Antigen retrieval was performed in a pH 6.0 citrate buffer solution in an oven for 20 min, followed by 20 min of cooling on the benchtop. Endogenous peroxidase activity was quenched in a 3% hydrogen peroxide solution (1.08600, Sigma) for 30 min. Subsequently, IHC staining was carried out using an anti-CAPRIN1 antibody (ab244360, Abcam, UK). After antibody incubation, sections were treated with rabbit secondary antibody labeled with HRP (ab6721, Abcam) and visualized using DAB substrate (ab64238, Abcam) for color development. Sections were then observed and analyzed under a light microscope to determine the percentage of positively stained cells.

### Hematoxylin and eosin (H&E) staining

Tissue samples were fixed in 4% paraformaldehyde (Sigma-Aldrich, USA) for 30–50 min, dehydrated overnight, embedded in Technovit 7100 glycol methacrylate (Heraeus Kulzer GmBH, Wehrheim, Germany), and sectioned to a thickness of 5 μm. H&E staining was carried out using the staining kit ab245880 (Abcam) following the manufacturer’s instructions. Subsequently, the stained sections were examined for tissue pathological features under a Nikon optical microscope.

### Flow cytometry analysis

Tumor tissues from various treatment groups were collected for analysis. The cell suspensions were washed with PBS and centrifuged at 500×g for 5 min at 4 °C. The supernatant was carefully removed from the pellets. The enriched cells were resuspended in 50 µl of cell staining buffer (CSB) (420201, Biolegend, USA) containing 10% FcR-Block (130-059-901, Miltenyi), followed by an incubation at 4 °C for 15 min. For staining, the cells were incubated with Anti-CD8-FITC (ab95591, abcam), Anti-CD25-APC (ab267381, abcam), and Anti-CD4-FITC (ab59474, abcam) in a final volume of 100 µl per sample, which included CSB and 50 µl of Brilliant staining buffer (563794, BD) at 4 °C. After washing the cells with 2 ml of CSB and resuspending them in 500 µl of PBS, cell sorting was performed using FACSAria Fusion (BD).

### Statistical analysis methods

The data were obtained from at least three independent experiments, and the results were presented as mean ± standard deviation (Mean ± SD). For comparisons between two groups, the independent samples t-test was utilized. When comparing three or more groups, one-way analysis of variance (ANOVA) was employed. If the ANOVA results indicated significant differences, post hoc Tukey’s Honestly Significant Difference (HSD) test was conducted to compare the differences among groups. In cases of non-normal distribution or heteroscedasticity of data, the Mann-Whitney U test or the Kruskal-Wallis H test would be used. All statistical analyses were performed using GraphPad Prism 9 (GraphPad Software, Inc.) and R programming language. The significance level for all tests was set at 0.05, and a two-tailed *p*-value less than 0.05 was considered statistically significant.

## Results

### Participation of LINC02544/miR-497-5p in TNBC progression

**Fig. 1:**
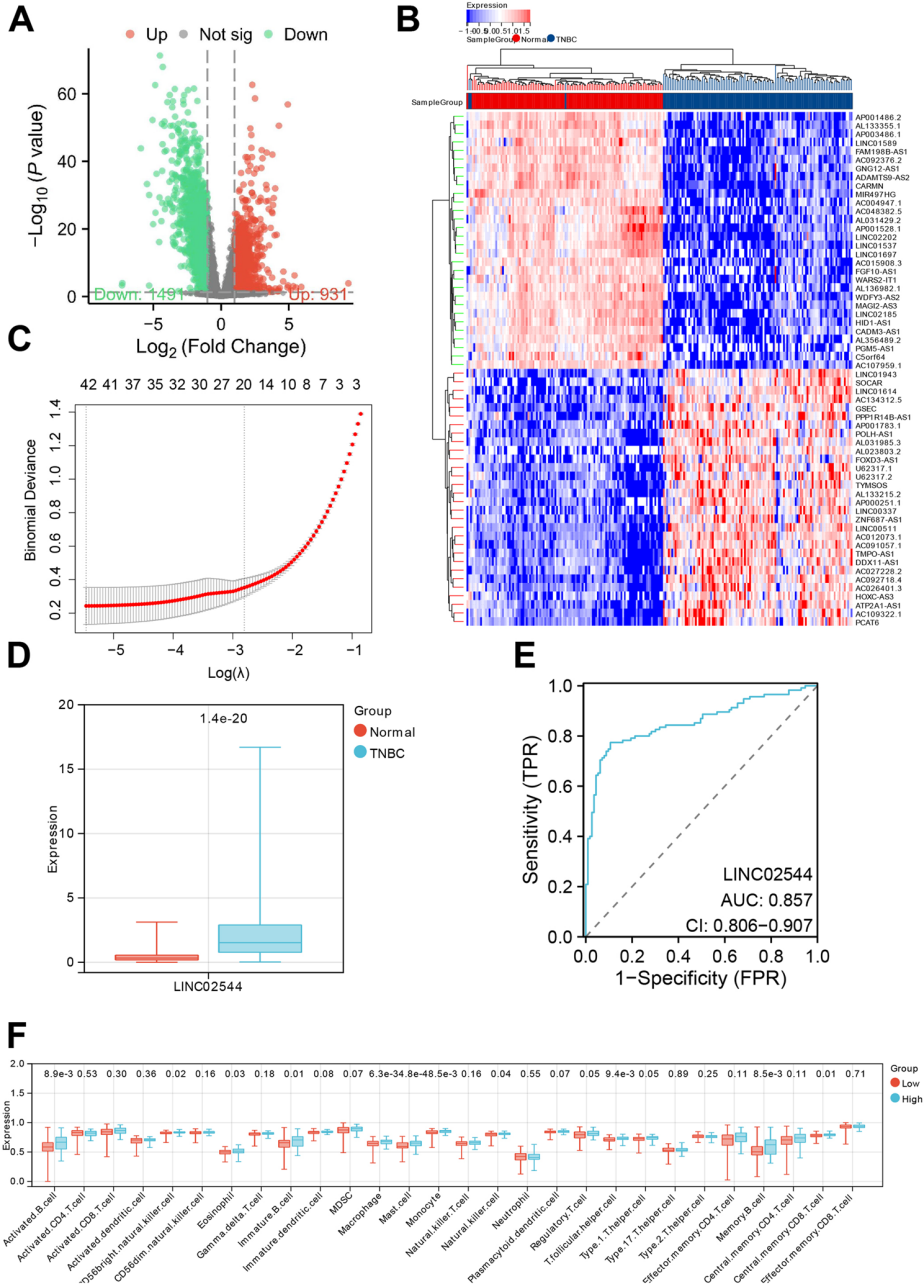
Analysis of LINC02544 Expression Patterns and Biological Significance in TNBC. Note: (**A**) Volcano plot of differential lncRNA analysis in the TCGA-TNBC dataset; red indicates significantly upregulated genes, green indicates significantly downregulated genes, and gray indicates non-significant genes. (**B**) Heatmap of the top 30 genes. (**C**) LASSO coefficient screening plot. (**D**) Expression of LINC02544 in the TCGA-TNBC dataset. (**E**) ROC curve of LINC02544 in the TCGA-TNBC dataset; Y-axis represents sensitivity, X-axis represents specificity. (**F**) Boxplot comparing the infiltration of 28 cell types between high and low LINC02544 expression groups; Low: *n* = 57, High: *n* = 58. Normal: *n* = 113, TNBC: *n* = 115

We downloaded the BRCA dataset from the TCGA database and selected 116 TNBC samples based on clinical data, along with 113 normal samples, to form the TCGA-TNBC dataset. Functionally, lncRNAs are involved in gene expression, subcellular transport, protein degradation, and organelle biogenesis (Mattick et al. 2009). Research indicates that abnormal expression of lncRNAs significantly regulates TNBC cell proliferation, migration, metastasis, and tumorigenicity (Xu et al. 2020). From the TCGA-TNBC dataset, we obtained the lncRNA expression profile, which includes 115 TNBC samples and 113 normal samples. Differential analysis using the “LIMMA” package identified 2,422 DEGs, with 1,491 significantly downregulated and 931 significantly upregulated DEGs (Fig. 1A-B).

We further conducted multivariable Cox analysis using LASSO regression on these 2,422 DEGs, resulting in 42 DEGs (Fig. 1C). Among these, we focused on LINC02544, which has been reported as a potential biomarker and therapeutic target for breast cancer patients undergoing neoadjuvant therapy (Guo et al. 2020). LINC02544 is significantly overexpressed in the TCGA-TNBC dataset (Fig. 1D), and ROC curve analysis shows an AUC value of 0.857, indicating high diagnostic efficiency in distinguishing TNBC from normal samples (Fig. 1E).

Using the ssGESA package in R, we analyzed the infiltration of 28 cell types in the TCGA-TNBC dataset. We divided the 115 TNBC samples into high and low expression groups based on LINC02544 expression. The high expression group exhibited greater infiltration of immune cells, including mast cells, monocytes, and activated B cells (Fig. 1F).

**Fig. 2:**
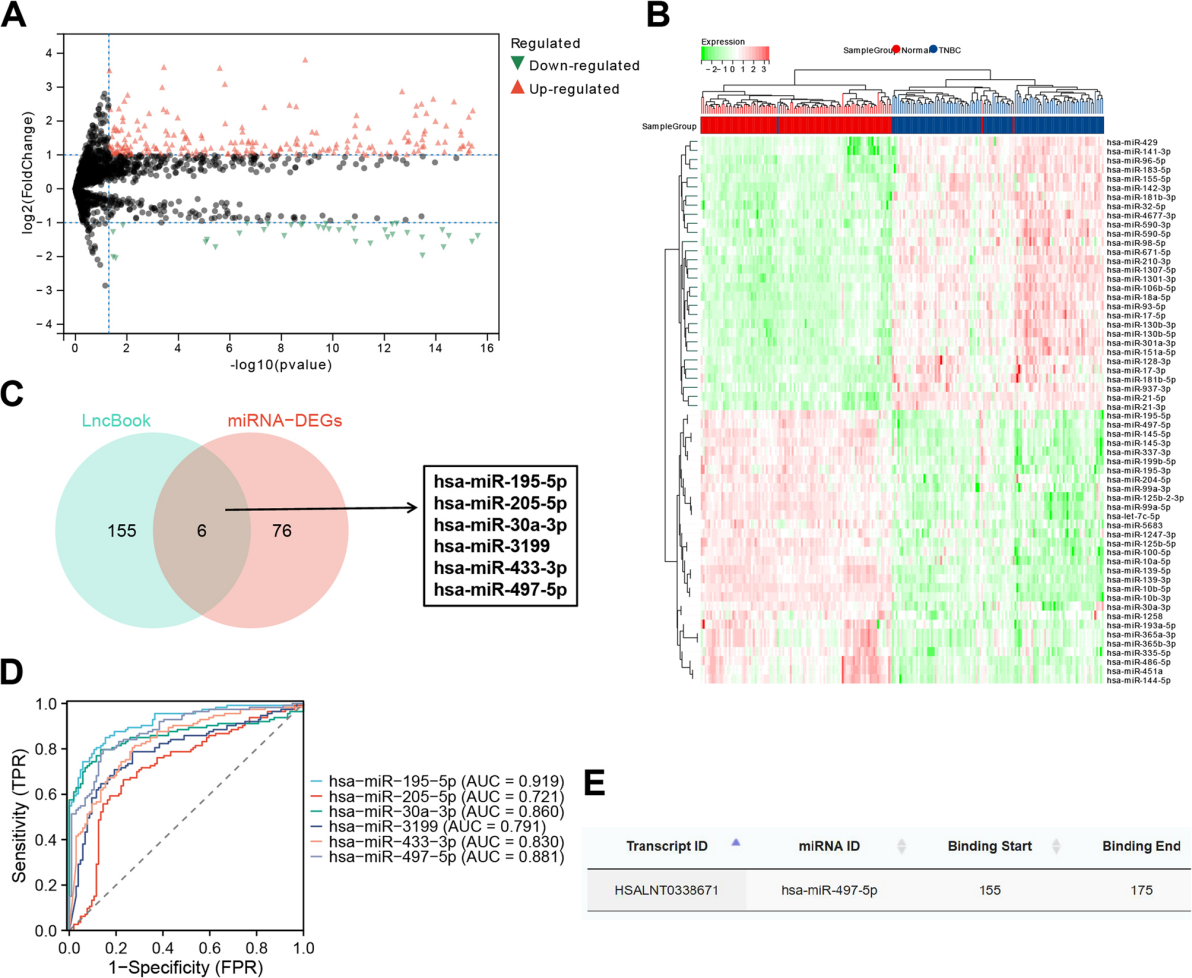
Exploration of miRNA Targets of LINC02544 in the TCGA-TNBC Dataset. Note: (**A**) Volcano plot of differential miRNA analysis in the TCGA-TNBC dataset; red indicates significantly upregulated genes, blue indicates significantly downregulated genes, and gray indicates non-significant genes. (**B**) Heatmap of the top 30 miRNAs. (**C**) Venn diagram of LINC02544 miRNA targets annotated by the LncBook dataset intersecting with significantly downregulated miRNAs. (**D**) ROC curves of six intersecting miRNAs in the TCGA-TNBC dataset; Y-axis represents sensitivity, X-axis represents specificity. (**E**) Binding relationship between LINC02544 and miR-497-5p in the LncBook dataset. Normal: *n* = 104; TNBC: *n* = 113

In the previous analysis, we identified LINC02544 as significantly overexpressed in TNBC and influential in the infiltration of various immune cells. Next, we sought to identify downstream miRNAs. We first obtained the miRNA expression matrix from the TCGA-TNBC dataset, which includes 113 TNBC samples and 104 normal samples, and conducted differential analysis. This analysis identified 389 significantly differentially expressed miRNAs, with 82 significantly downregulated and 307 significantly upregulated (Fig. 2A-B).

Using the LncBook dataset, we annotated 161 miRNA targets of LINC02544 and intersected these with the significantly downregulated miRNAs, resulting in 6 intersecting miRNAs (Fig. 2C). ROC curve analysis showed that miR-195-5p, miR-30a-3p, and miR-497-5p had AUC values of 0.919, 0.860, and 0.881, respectively, indicating high diagnostic efficiency (Fig. 2D). Data suggests that restoring miR-497-5p can inhibit tumor PD-L1 expression, suppress tumor proliferation and migration, and regulate various oncogenic signaling pathways in TNBC cells (Shadbad et al. 2021). Figure 2E illustrates the binding relationship between LINC02544 and miR-497-5p from the LncBook dataset.

In summary, our analysis highlights the importance of LINC02544 in TNBC, its correlation with immune cell infiltration, and predicts miR-497-5p as a miRNA target of LINC02544.

### Analysis of cellular heterogeneity and pathway activity reveals key molecular dynamics in TNBC

Our analysis reveals that LINC02544 in the TCGA-TNBC dataset regulates the infiltration of various immune cell types and the expression of miR-497-5p. To further explore related target genes, we downloaded the TNBC scRNA-seq dataset GSE221743 from the GEO database, which includes a TNBC tumor sample. Using the Seurat package, we integrated the data and examined the number of genes (nFeature_RNA), the number of mRNA molecules (nCount_RNA), and the percentage of mitochondrial genes (percent.mt) in all cells (Figure S1A). The results showed that most cells had nFeature_RNA < 5000, nCount_RNA < 20,000, and percent.mt < 20% (Figure S1B). We filtered out low-quality cells using the criteria of 200 < nFeature_RNA < 5000 and percent.mt < 20%.

The correlation analysis of sequencing depth indicated that the filtered data had a correlation coefficient *r* = −0.15 between nCount_RNA and percent.mt, and *r* = 0.95 between nCount_RNA and nFeature_RNA (Figure S1C), demonstrating the high quality of the filtered cell data, suitable for further analysis.

Next, we analyzed the filtered cells. Using the ElbowPlot method, we ranked the PCs by standard deviation, finding that PC_1– PC_20 effectively reflected the information contained in the highly variable genes, providing significant analytical value (Figure S1D). We then applied the UMAP algorithm for nonlinear dimensionality reduction on the top 20 PCs and displayed clustering results at different resolutions using the clustree package (Figure S1E). UMAP clustering analysis grouped all cells into 19 clusters (Figure S1F).

**Fig. 3:**
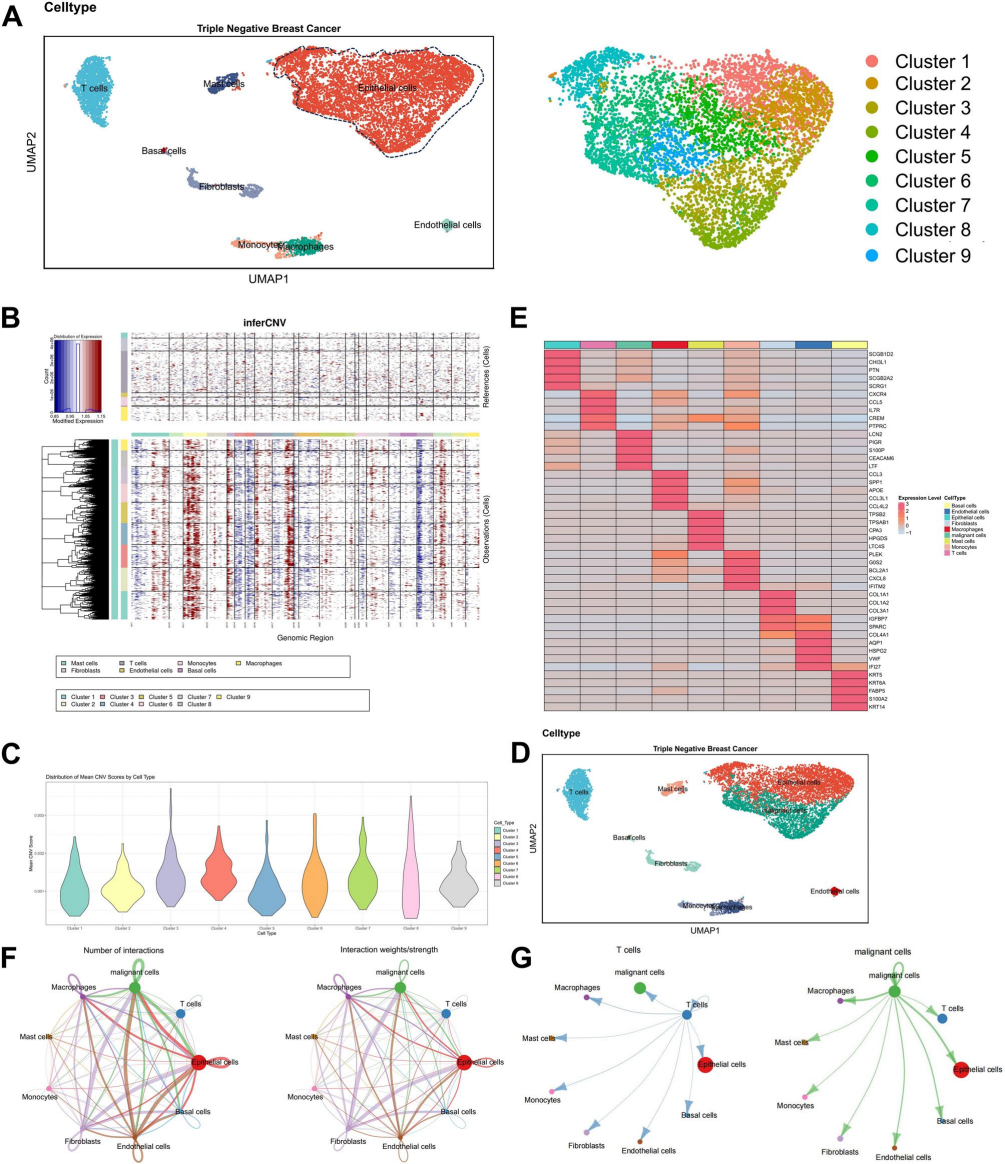
scRNA-seq Reveals Molecular Characteristics and Interactions of TNBC Cell Populations. Note: (**A**) Left: UMAP visualization of cell annotation results based on clustering; Right: Extraction and annotation of epithelial cells into nine clusters. (**B**) InferCNV analysis showing CNV expansions and deletions, with mast cells, basal cells, endothelial cells, macrophages, fibroblasts, monocytes, and T cells as controls. (**C**) Violin plot of CNV scores for the nine epithelial cell clusters. (**D**) UMAP visualization of nine re-annotated cell types after identifying malignant cells. (**E**) Heatmap of the top five genes for the nine cell types. (**F**) Circos plot of cell communication in the sample, with line thickness representing the number of pathways (left) and interaction strength (right). (**G**) Detailed display of communication between malignant epithelial cells and other cell types. TNBC: *n* = 1

Based on a review of relevant literature and using the online resource CellMarker to identify marker genes for various human cell types, we employed the Bioconductor/R package “SingleR” for automatic annotation. This analysis identified eight cell types, and the distribution of marker genes confirmed the accuracy of the annotations (Figure S1G). The identified cell types include mast cells, basal cells, epithelial cells, endothelial cells, macrophages, fibroblasts, monocytes, and T cells, with epithelial cells comprising nine clusters (Fig. 3A).

Using the InferCNV tool, we analyzed chromosome CNVs in epithelial cells. We found that clusters 3, 4, 7, and 9 had higher CNV scores, defining these clusters as malignant cells (Fig. 3B-D). A heatmap showing the top five genes for each of the nine cell types demonstrated the reliability of the results (Fig. 3E).

We employed the R package “CellChat” to investigate pathway activity between different cell types. This analysis revealed interactions among the nine cell types, showing that malignant cells have a higher correlation with fibroblasts and macrophages compared to other immune cells (Fig. 3F-G).

Through the analysis of the scRNA-seq dataset, we conducted a detailed study of cellular heterogeneity in TNBC samples and explored the interactive pathway activities between malignant cells and other cell types.

### Revealing the critical role of the LINC02544/miR-497-5p/CAPRIN1 axis in TNBC progression

**Fig. 4:**
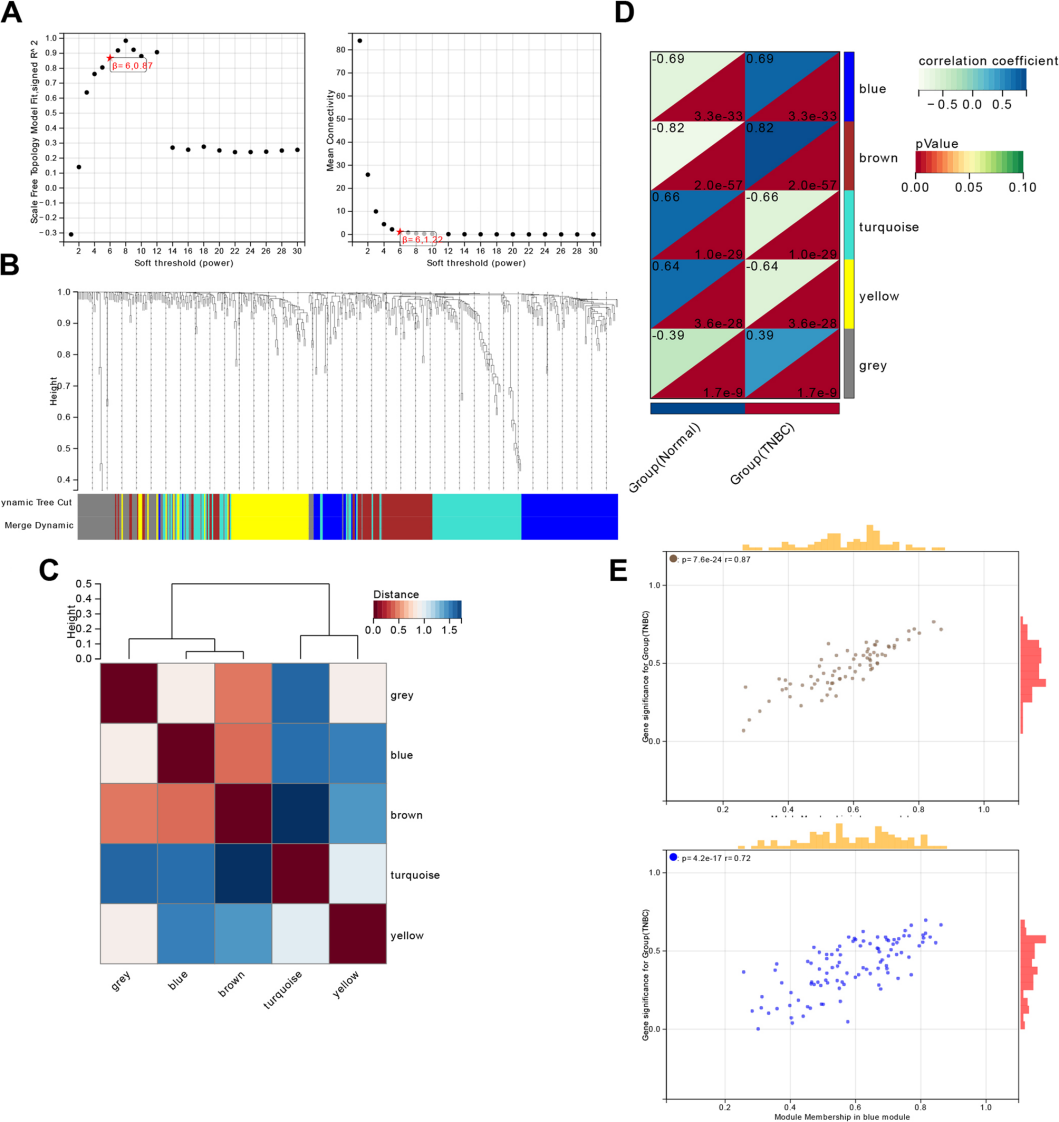
WGCNA Analysis Based on scRNA-seq Combined with TCGA-TNBC Data. Note: (**A**) Scale independence (left) and mean connectivity (right) for determining the weighted value β = 6 that meets the scale-free network law. (**B**) Dendrogram of co-expression network modules. (**C**) Heatmap of correlations among the six modules. (**D**) Correlation analysis between modules and the TNBC group. (**E**) Scatter plot analysis of the brown and blue modules with the TNBC group

Through the analysis of the TNBC scRNA-seq dataset, we successfully annotated malignant cells and identified significant interactions between these malignant cells and various other cell types via cell communication analysis. Using the WGCNA algorithm, we constructed a co-expression network of characteristic genes in 838 malignant epithelial cells, selecting β = 6 to satisfy the scale-free network law. Under the parameters of minimum module size = 30 and module merging threshold = 0.25, we identified six modules (Fig. 4A), as shown in the clustering dendrogram (Fig. 4B). The correlations among these six modules are presented in Fig. 4C.

We then used the Normal and TNBC groups as clinical traits to re-screen gene modules significantly associated with TNBC. The results indicated that the brown and blue modules were most significantly correlated with TNBC, both showing positive correlations (Fig. 4D-E). Therefore, we selected the genes from the brown and blue modules, totaling 175 genes, as significantly associated with TNBC.

**Fig. 5:**
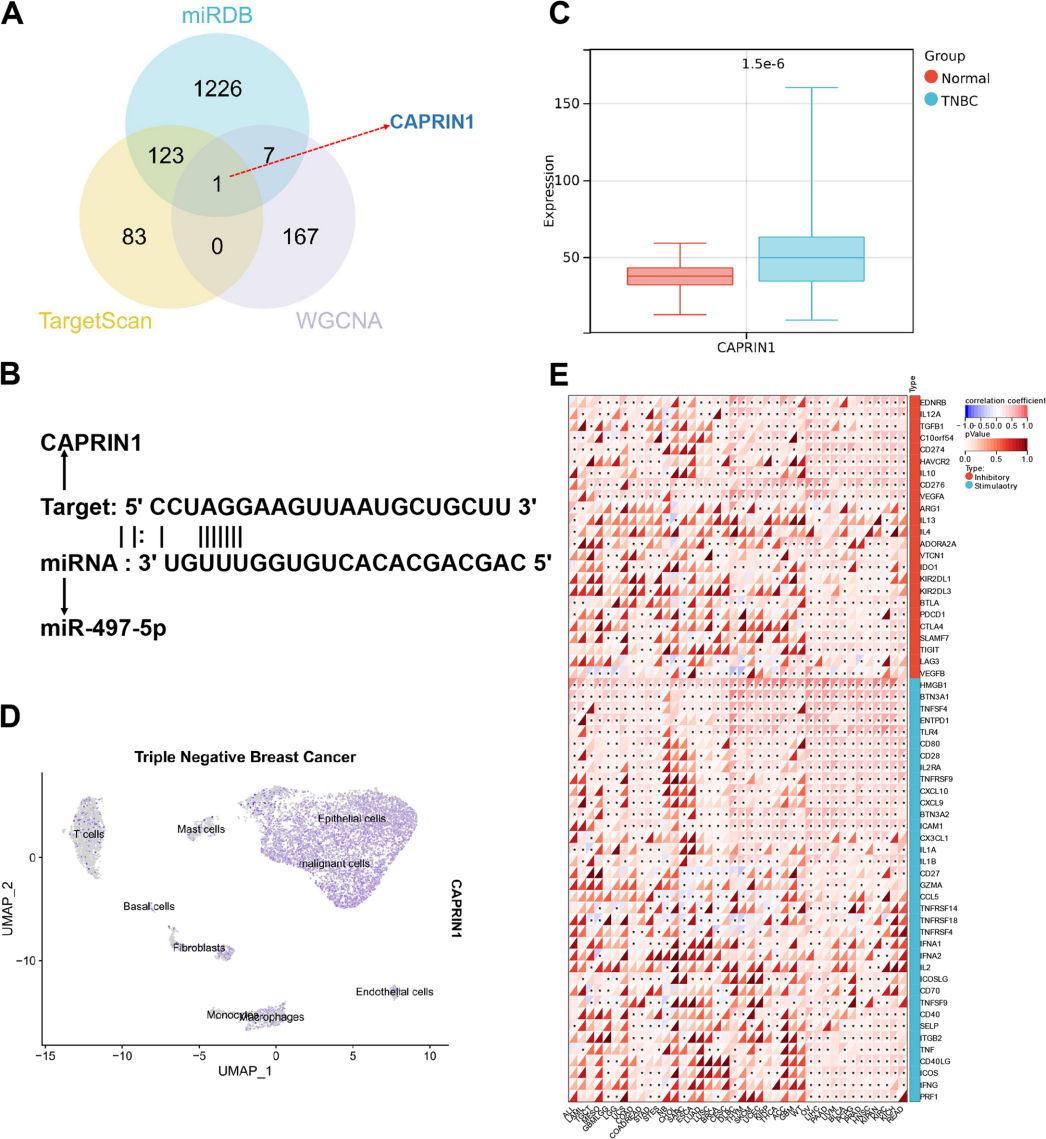
Exploring the Impact of the LINC02544/miR-497-5p/CAPRIN1 Axis on TNBC Regulatory Mechanisms. Note: (**A**) Venn diagram of miR-497-5p mRNA targets predicted by miRDB and TargetScan datasets intersecting with TNBC-related genes from two WGCNA modules. (**B**) Verification of the interaction between miR-497-5p and CAPRIN1 using the StarBase database. (**C**) Boxplot of CAPRIN1 expression in the TCGA-TNBC dataset; Normal: *n* = 113, TNBC: *n* = 115. (**D**) UMAP plot of CAPRIN1 expression in the scRNA-seq dataset; TNBC, *n* = 1. (**E**) Heatmap of correlations between CAPRIN1 and immune checkpoint genes in pan-cancer analysis, with * indicating *p* < 0.05; sample size = 19,131

Next, we aimed to identify key miRNA targets by predicting miR-497-5p mRNA targets using the miRDB and TargetScan databases. The miRDB database annotated 1,357 target mRNAs, and the TargetScan database annotated 207 target mRNAs. Intersecting the miRNA datasets with the TNBC-related genes from the two modules, we identified CAPRIN1 as the sole intersecting gene (Fig. 5A).

We further validated the interaction between miR-497-5p and CAPRIN1 using the StarBase database (https://rnasysu.com/encori/) (Fig. 5B). CAPRIN1 has been reported to be involved in the progression of various cancers (Liu et al. 2021a, b; Zhang et al. 2018; (Shi, et al., 2019)). In our selected datasets, CAPRIN1 was significantly upregulated in TCGA-TNBC (Fig. 5C) and predominantly expressed in epithelial and malignant cells in the scRNA-seq dataset (Fig. 5D).

Additionally, in the pan-cancer datasets (TCGA, TARGET, and GTEx), CAPRIN1 showed significant correlations with 60 immune checkpoints (Fig. 5E).

This study comprehensively utilized TCGA-TNBC and scRNA-seq data and molecular network analysis methods to explore the mechanism of the LINC02544/miR-497-5p/CAPRIN1 axis in TNBC development. Moreover, it revealed the relationship between CAPRIN1 and immune checkpoints.

### Knockdown of LINC02544 activates miR-497-5p and inhibits CAPRIN1 expression

**Fig. 6:**
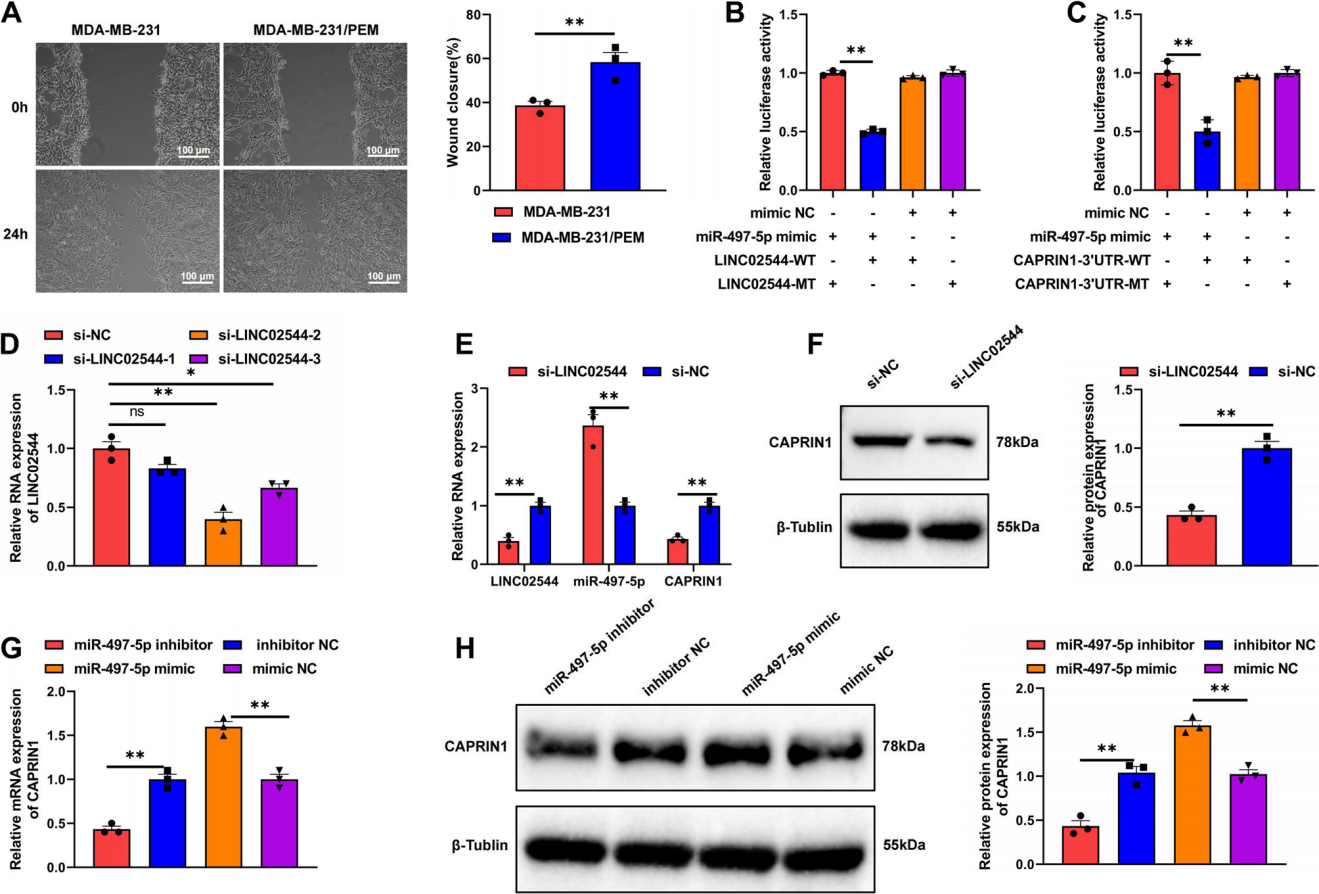
Effects of LINC02544 Knockdown on CAPRIN1 Expression and miR-497-5p Activity. Note: (**A**) Scratch assay comparing the migration ability of MDA-MB-231/PEM and MDA-MB-231 cells treated with 10 µg PEM. (**B**) Dual-luciferase assay showing the relative expression of LINC02544 binding site WT and MT in HEK293T cells overexpressing miR-497-5p and control groups. (**C**) Dual-luciferase assay showing the relative expression of CAPRIN1 3’UTR binding site WT and MT in HEK293T cells overexpressing miR-497-5p and control groups. (**D**) qPCR analysis of the knockdown efficiency of three si-LINCO2544. (**E**) qPCR analysis of the relative expression levels of LINC02544, miR-497-5p, and CAPRIN1 in each group. (**F**) WB analysis of the relative protein expression levels of CAPRIN1 in each group. (**G**) qPCR analysis of the relative expression levels of CAPRIN1 in each group. (**H**) WB analysis of the relative protein expression levels of CAPRIN1 in each group. All cell experiments were repeated three times. ns indicates *p* > 0.05, *p < 0.05, **p < 0.01

To verify whether LINC02544 regulates through the miR-497-5p/CAPRIN1 axis in immune-resistant TNBC cell line MDA-MB-231/PEM, we treated MDA-MB-231/PEM and MDA-MB-231 cells with 10 µg/ml Pembrolizumab. A scratch assay was performed to measure cell proliferation at 0 h and 24 h. The results indicated that the scratch healing rate of MDA-MB-231/PEM cells was faster than that of MDA-MB-231 cells, confirming the successful construction of immune-resistant MDA-MB-231/PEM cells (Fig. 6A).

To determine if LINC02544 binds to miR-497-5p, we conducted a dual-luciferase reporter assay. HEK293T cells were co-transfected with wild-type (WT) or mutant-type (MT) reporter vectors containing the LINC02544 binding sites. The results showed that overexpression of miR-497-5p significantly reduced the luciferase activity in WT constructs containing LINC02544 binding sites, but not in mutant (MT) constructs, indicating a direct interaction between LINC02544 and miR-497-5p (Fig. 6B). Further analysis revealed that overexpression of miR-497-5p significantly decreased the luciferase activity of WT constructs containing the CAPRIN1 3’UTR region, confirming the targeting of CAPRIN1 by miR-497-5p (Fig. 6C).

qPCR was used to assess the interference efficiency of three siRNAs targeting LINC02544 in MDA-MB-231/PEM cells. The si-LINC02544-2 sequence showed the highest knockdown efficiency and was selected for subsequent experiments, referred to as si-LINC02544 (Fig. 6D). RT-qPCR results demonstrated that knockdown of LINC02544 significantly decreased its expression, while significantly increasing miR-497-5p expression and decreasing CAPRIN1 expression in MDA-MB-231/PEM cells (Fig. 6E). WB analysis further validated these changes, showing a significant reduction in CAPRIN1 protein levels following LINC02544 knockdown in MDA-MB-231/PEM cells (Fig. 6F).

Additionally, experiments with miR-497-5p overexpression and knockdown in MDA-MB-231/PEM cells demonstrated that overexpression of miR-497-5p significantly reduced both mRNA and protein levels of CAPRIN1, while miR-497-5p knockdown significantly increased CAPRIN1 mRNA and protein levels (Fig. 6G-H).

These results suggest that LINC02544 promotes CAPRIN1 expression by inhibiting miR-497-5p. Additionally, we further validated the expression patterns of LINC02544, miR-497-5p, and CAPRIN1 in MDA-MB-231 and MDA-MB-231/PEM cells by RT-qPCR. The results showed that LINC02544 and CAPRIN1 expression levels were significantly elevated, while miR-497-5p expression was markedly decreased in resistant cells compared to parental cells (Figure S2).

### Successful loading and efficient uptake of fluorescently labeled si-LINCO2544 into exos

Exos, as specifically secreted vesicles, play a role in intercellular communication. Research has shown that exos can function as effective carriers with anti-tumor effects on glioblastoma (Kim et al. 2020). Recent studies also reported that exos can deliver YY1 siRNA to glioblastoma cells, effectively releasing their payload and enhancing chemotherapy sensitivity (Liu et al. 2022). To efficiently target and deliver LINC02544 siRNA, we extracted exos from the MDA-MB-231 TNBC cell line using ultracentrifugation. We then loaded FAM-labeled si-LINCO2544 into the exos via electroporation.

**Fig. 7:**
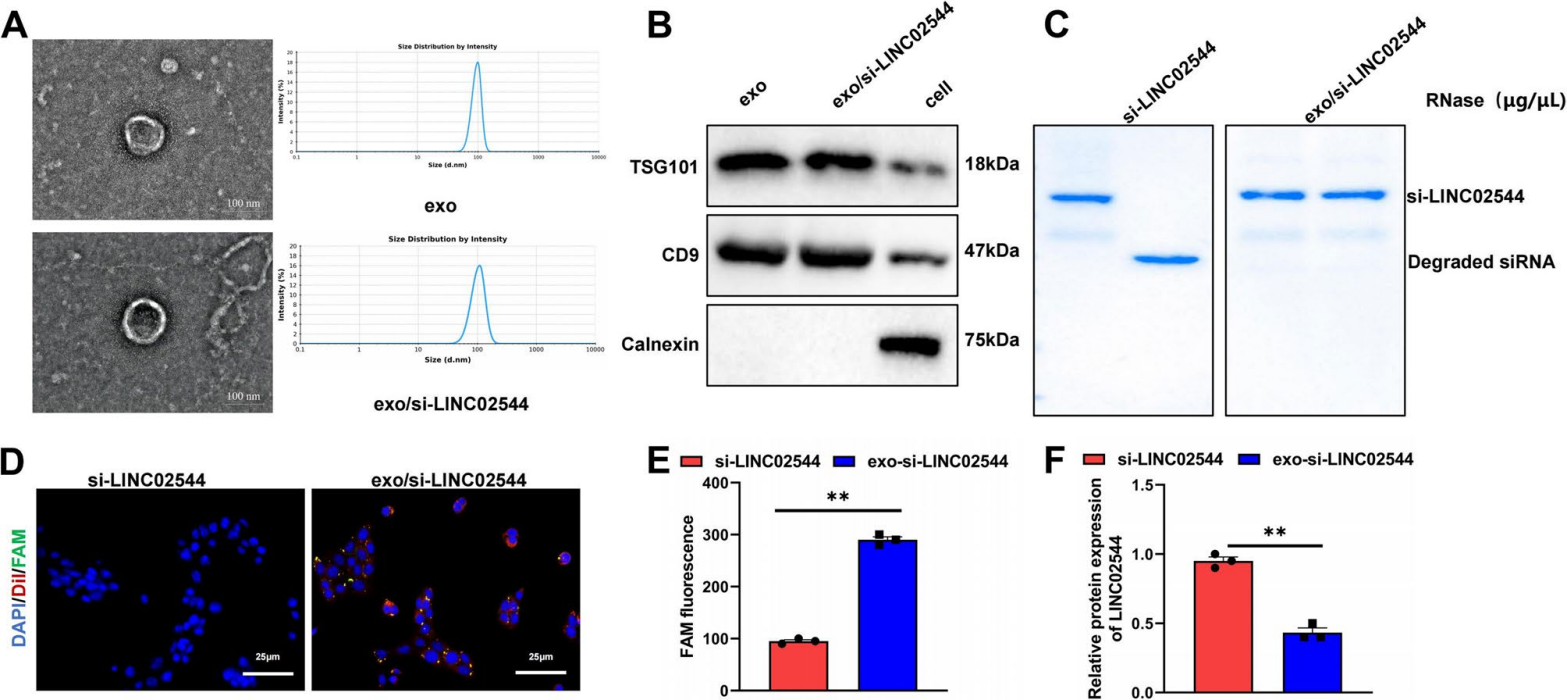
Loading and Uptake Efficiency of Fluorescently Labeled si-LINCO2544 in the Exo Carrier System. Note: (**A**) NTA characterizing exosomes and exo/si-LINC02544 extracted from MDA-MB-231/PEM cells, with TEM images confirming the structure of exosomes and exo/si-LINC02544. (**B**) WB analysis of exosome markers CD9 and TSG101, and the endoplasmic reticulum membrane protein Calnexin. (**C**) Stability of RNA after RNase A treatment of si-LINC02544 and exo/si-LINC02544. (**D**) Fluorescence microscopy showing successful incorporation of fluorescently labeled si-LINCO2544 into exosomes using electroporation and its efficient uptake by cells; blue indicates DAPI (nucleus), red indicates Dil (exosome), and green indicates FAM (si-LINC02544). Scale bar: 25 μm. (**E**) Flow cytometry analysis of siRNA exosome uptake efficiency. (**F**) qPCR analysis of LINC02544 expression changes after exo/si-LINC02544 and PBS treatment. All cell experiments were repeated three times, **p < 0.01

NTA indicated that the size distribution of exos and exo/si-LINC02544 ranged between 50 and 180 nm, with smooth NTA curves and no significant differences between the two groups. TEM confirmed the morphology of the exos, showing that both exos and exo/si-LINC02544 were round or oval-shaped vesicles, with no apparent differences between them (Fig. 7A).

WB analysis detected surface marker proteins CD9 and TSG101, and the endoplasmic reticulum membrane protein Calnexin in exos, exo/si-LINC02544, and cell lysates. The results showed high expression of CD9 and TSG101 in the exo samples, while Calnexin was absent in the exo samples but highly expressed in the cell lysates, confirming the purity and quality of the exos (Fig. 7B).

To assess the stability of si-LINC02544 in exos, we treated si-LINC02544 and exo/si-LINC02544 with RNase A. The results demonstrated that si-LINC02544 was completely degraded, whereas exo/si-LINC02544 remained largely intact (Fig. 7C). Fluorescence microscopy showed successful loading of fluorescently labeled siRNA into exos and efficient uptake by MDA-MB-231/PEM cells (Fig. 7D). Flow cytometry further validated the uptake efficiency, showing a significant increase in fluorescence intensity in treated cells (Fig. 7E). Additionally, the expression of LINC02544 in MDA-MB-231/PEM cells was significantly reduced in the presence of exo/si-LINC02544 compared to PBS treatment (Fig. 7F).

These results indicate that the strategy of constructing tumor cell exo/si-LINC02544 is successful, with the exo/si-LINC02544 demonstrating good uptake efficiency and stability in vitro.

### Exo/si-LINC02544 inhibit proliferation and migration of TNBC cells

**Fig. 8:**
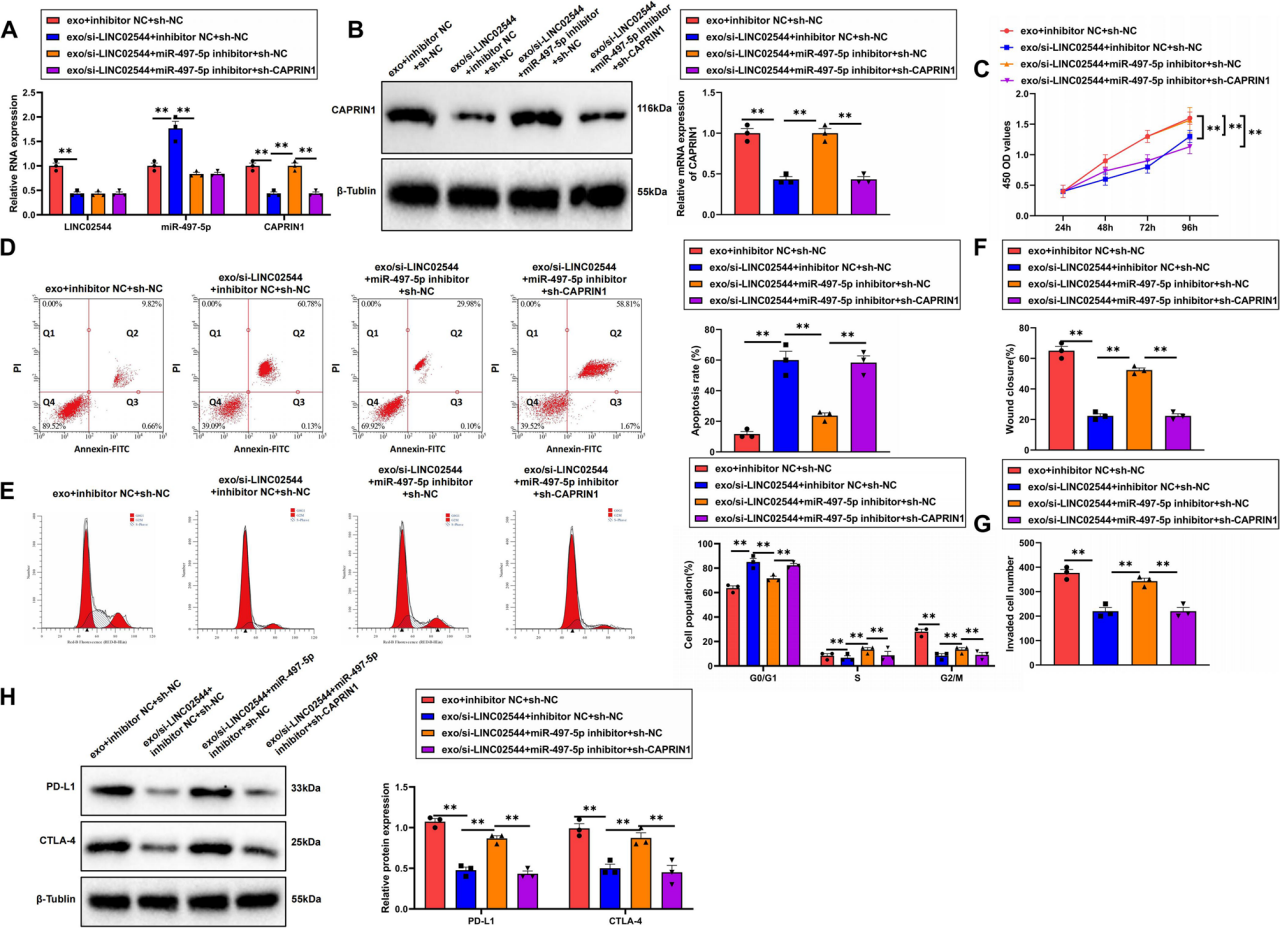
Inhibitory Effects of Exo/si-LINC02544 on TNBC Cell Proliferation and Migration. Note: (**A**) RT-qPCR analysis of LINC02544, miR-497-5p, and CAPRIN1 expression levels in MDA-MB-231/PEM cells. (**B**) WB analysis of CAPRIN1 expression in MDA-MB-231/PEM cells. (**C**) CCK8 assay measuring cell viability in MDA-MB-231/PEM cells. (**D**) Flow cytometry analysis of apoptosis changes in MDA-MB-231/PEM cells. (**E**) Flow cytometry analysis of cell cycle changes in MDA-MB-231/PEM cells. (**F**) Scratch assay results revealing changes in cell migration ability in MDA-MB-231/PEM cells. (**G**) Transwell invasion assay results showing changes in cell invasion ability in MDA-MB-231/PEM cells. (**H**) WB analysis of immune checkpoint proteins PD-L1 and CTLA-4 expression in MDA-MB-231/PEM cells. All cell experiments were repeated three times; **p < 0.01

To investigate the effects of exo/si-LINC02544 on the immune-resistant TNBC cell line MDA-MB-231/PEM via the miR-497-5p/CAPRIN1 axis, we first constructed the immune-resistant MDA-MB-231/PEM cell line. RT-qPCR analysis revealed that treatment with exo/si-LINC02544 significantly decreased LINC02544 expression, upregulated miR-497-5p expression, and downregulated CAPRIN1 expression. Conversely, treatment with miR-497-5p inhibitors resulted in no significant change in LINC02544 expression, a significant decrease in miR-497-5p expression, and an increase in CAPRIN1 expression. Additionally, sh-CAPRIN1 treatment did not significantly affect LINC02544 and miR-497-5p expression but significantly decreased CAPRIN1 expression (Fig. 8A). WB analysis corroborated these findings, showing a significant reduction in CAPRIN1 protein levels in the exo/si-LINC02544 treatment group, which increased with miR-497-5p inhibitor treatment and decreased with sh-CAPRIN1 treatment (Fig. 8B).

CCK-8 assay results indicated that exo/si-LINC02544 significantly inhibited the proliferation of resistant cells. In contrast, miR-497-5p inhibitor treatment significantly promoted proliferation, whereas sh-CAPRIN1 treatment significantly inhibited proliferation (Fig. 8C). Flow cytometry analysis showed that exo/si-LINC02544 significantly promoted apoptosis in resistant cells, while miR-497-5p inhibitor treatment significantly inhibited apoptosis, and sh-CAPRIN1 treatment significantly promoted apoptosis (Fig. 8D). Moreover, cell cycle analysis revealed that exo/si-LINC02544 increased the proportion of cells in the G0/G1 phase and decreased the proportion in the S phase. In contrast, miR-497-5p inhibitor treatment decreased the G0/G1 phase proportion and increased the S phase proportion, while sh-CAPRIN1 treatment increased the G0/G1 phase proportion and decreased the S phase proportion (Fig. 8E).

To determine whether exo/si-LINC02544 affect the invasion and migration of TNBC cells, we conducted scratch and Transwell assays. The results indicated that treatment with exo/si-LINC02544 significantly inhibited the migration and invasion capabilities of resistant cells. Conversely, treatment with miR-497-5p inhibitors significantly promoted these capabilities, while sh-CAPRIN1 treatment significantly inhibited them (Fig. 8F-G). Previous bioinformatics analysis revealed a significant correlation between CAPRIN1 and various immune checkpoints. To verify whether si-LINCO2544 mediates the miR-497-5p/CAPRIN1 axis and regulates the expression of immune checkpoint-related factors, we performed WB analysis to assess the expression of specific immune checkpoint factors PD-L1 (programmed death-ligand 1) and CTLA-4 (cytotoxic T-lymphocyte-associated protein 4). The results showed that si-LINCO2544 treatment significantly decreased the expression levels of PD-L1 and CTLA-4. In contrast, miR-497-5p inhibitor treatment significantly increased the expression levels of these factors, while sh-CAPRIN1 treatment significantly decreased their expression levels (Fig. 8H). These findings suggest that exo/si-LINC02544 can significantly inhibit the proliferation and migration of TNBC cells by regulating the LINC02544/miR-497-5p/CAPRIN1 axis.

### Exo/si-LINC02544 combined with PD-L1 inhibitors significantly suppress breast cancer growth

To further validate the effect of exo/si-LINC02544 on tumor growth in immune therapy-resistant TNBC xenograft models via the miR-497-5p/CAPRIN1 axis, we established immune therapy-resistant TNBC xenograft mice by injecting MDA-MB-231/PEM cells. The mice were treated with exo/si-LINC02544, Pembrolizumab, miR-497-5p inhibitors, and sh-CAPRIN1 lentivirus. All groups were treated with PEM, and tumor size was measured using calipers.

**Fig. 9:**
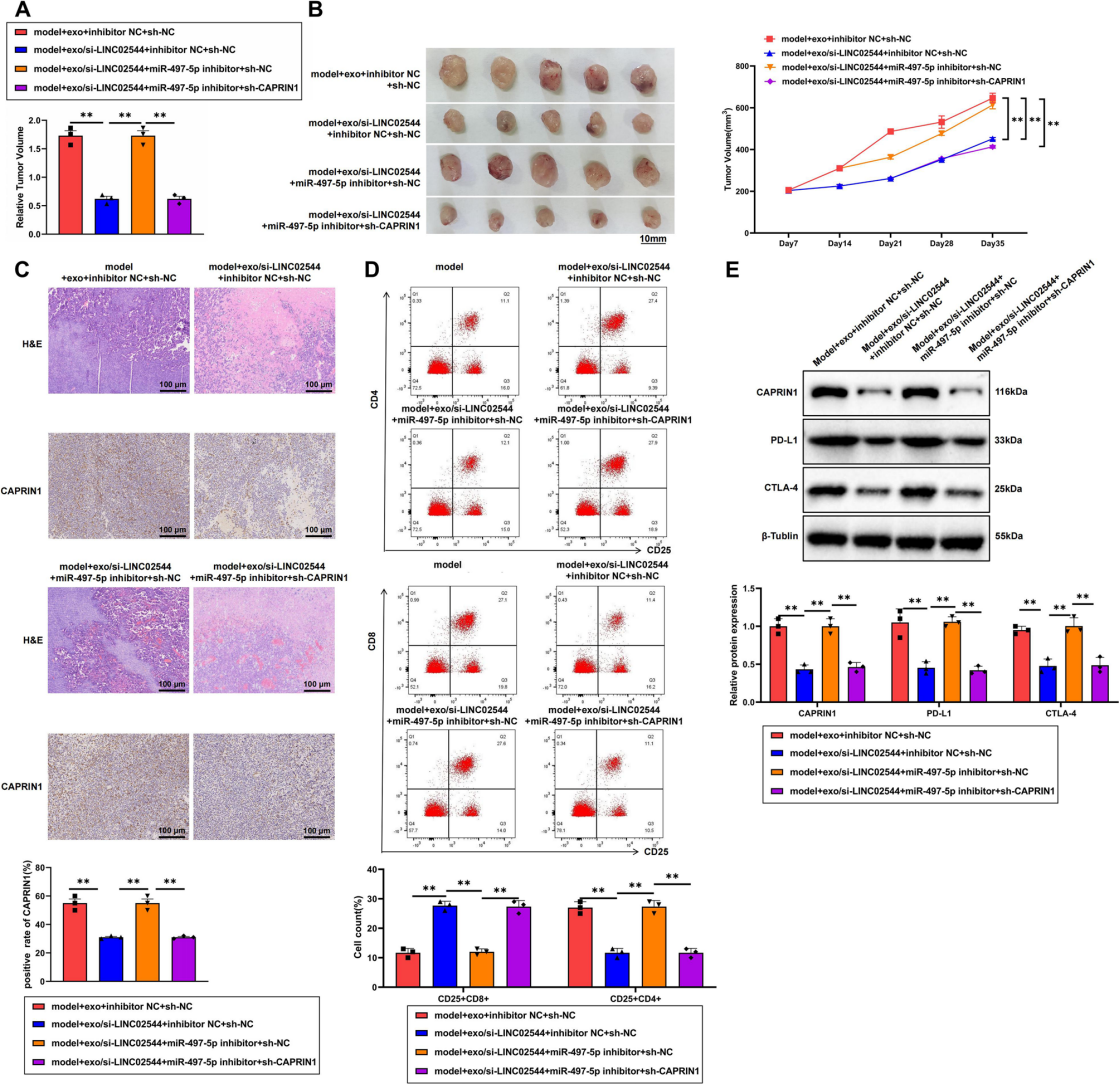
Combined Effects of Exo/si-LINC02544 and PD-L1 Inhibitors on TNBC Treatment. Note: (**A**) Tumor growth rate differences among the groups of mice, *N* = 6. (**B**) Photographic comparison of tumor sizes in each group of mice, *N* = 6. (**C**) H&E staining to assess tumor pathology and IHC to detect CAPRIN1-positive cells in each group; scale bar = 100 μm, *N* = 6. (**D**) Flow cytometry analysis of CD25 + CD8 + T cells and CD25 + CD4 + T cells, *N* = 6. (**E**) WB analysis of CAPRIN1, PD-L1, and CTLA-4 expression in tumor tissues of each group, *N* = 6, **p < 0.01

The results showed no significant differences in tumor growth rate and size between the inhibitor NC + sh-NC group and the control group. However, the mice treated with exo/si-LINC02544 exhibited a slower tumor growth rate. In contrast, the miR-497-5p inhibitor treatment accelerated tumor growth and increased tumor size, while sh-CAPRIN1 treatment slowed tumor growth and reduced tumor size (Fig. 9A-B).

H&E staining results revealed no significant differences in tumor pathology between the inhibitor NC + sh-NC group and the control group. In mice treated with exo/si-LINC02544, tumor cell density significantly decreased, and necrotic areas increased. Conversely, miR-497-5p inhibitor treatment resulted in a significant increase in tumor cell density and a decrease in necrotic areas, while sh-CAPRIN1 treatment led to a significant reduction in tumor cell density and an increase in necrotic areas.

IHC analysis showed no significant change in CAPRIN1 expression between the inhibitor NC + sh-NC group and the control group. However, CAPRIN1 expression significantly decreased in the tumors of mice treated with exo/si-LINC02544. In contrast, miR-497-5p inhibitor treatment significantly increased CAPRIN1 expression, while sh-CAPRIN1 treatment significantly decreased CAPRIN1 expression (Fig. 9C).

Previous bioinformatics analysis suggested that LINC02544 might influence TNBC progression by affecting immune cell infiltration. To validate whether LINC02544 impacts tumor growth by altering the immune cell composition in the tumor microenvironment, we performed flow cytometry to analyze immune cell populations within the tumor tissue. The results showed no significant differences in CD8 + T cells and Treg cells between the inhibitor NC + sh-NC group and the control group. However, mice treated with exo/si-LINC02544 exhibited a significant increase in CD8 + T cells and a significant decrease in Treg cells. Conversely, treatment with miR-497-5p inhibitors resulted in a significant decrease in CD8 + T cells and an increase in Treg cells. In contrast, sh-CAPRIN1 treatment significantly increased CD8 + T cells and decreased Treg cells in the tumor tissue (Fig. 9D).

Furthermore, WB analysis of tumor tissues from different treatment groups revealed no significant differences in the expression of CAPRIN1, PD-L1, and CTLA-4 between the inhibitor NC + sh-NC group and the control group. However, exo/si-LINC02544 treatment significantly reduced the expression levels of CAPRIN1, PD-L1, and CTLA-4. Conversely, miR-497-5p inhibitor treatment significantly increased the expression levels of these proteins, while sh-CAPRIN1 treatment resulted in a significant reduction in their expression (Fig. 9E).

These findings suggest that exo/si-LINC02544, combined with PD-L1 inhibitors, can significantly inhibit TNBC tumor growth, improve the immune microenvironment, and enhance the effectiveness of immunotherapy.

## Discussion

This study utilized scRNA-seq, transcriptomics, and bioinformatics analysis to elucidate the mechanism by which LINC02544 mediates immune therapy resistance in TNBC through the miR-497-5p/CAPRIN1 axis. Additionally, we explored the potential of exo/si-LINC02544 to reverse this resistance. TNBC is a significant challenge in breast cancer treatment due to its high invasiveness and lack of effective targeted therapies (Liu et al. 2022). Despite the significant advancements in immunotherapy across various cancers, the response rate in TNBC patients remains low (Liu et al. 2024). Therefore, this study not only provides new insights into the mechanisms of immune therapy resistance in TNBC but also lays the groundwork for developing new therapeutic strategies. This aligns with our research focus on other cancers, such as hepatocellular carcinoma and ovarian cancer, underscoring the importance of exploring molecular mechanisms and innovative therapies.

LINC02544, lncRNA, has been found to be aberrantly expressed in various cancers, but its specific role in TNBC remains unclear (Wei et al. 2022; Fan et al. 2022). Previous studies have shown that lncRNAs can regulate gene expression and influence cellular behavior through interactions with miRNAs (Wei et al. 2022; Fan et al. 2022). In this study, we analyzed data from the TCGA and GEO databases to identify the high expression of LINC02544 in TNBC. Experimental validation demonstrated that LINC02544 regulates the proliferation and migration of TNBC cells via the miR-497-5p/CAPRIN1 axis. While this function aligns with the roles of LINC02544 in other cancers, the specific mechanisms in TNBC require further exploration. Similar to our findings in hepatocellular carcinoma, the regulatory pathways of LINC02544 may vary across different cancers, necessitating additional research.

miR-497-5p, a critical miRNA, has been reported to regulate cell proliferation, migration, and invasion in various cancers (Jing et al. 2022; Zhang et al. 2022; Lu et al. 2023). Using annotations from the LncBook database and analysis of the TCGA-TNBC dataset, we identified miR-497-5p as a miRNA target of LINC02544 and experimentally validated the direct regulatory effect of LINC02544 on miR-497-5p. Further research revealed that miR-497-5p targets and regulates CAPRIN1, providing new insights into the mechanisms of immune therapy resistance in TNBC. This detailed analysis of miRNA function in cancer mirrors our approach in ovarian cancer research, offering new directions for uncovering complex regulatory networks.

CAPRIN1, an RNA-binding protein, plays a significant role in various physiological and pathological processes (Jia et al. 2022; Liang et al., 2023, Kurhade et al. 2023). Previous studies have highlighted the importance of CAPRIN1 in cell proliferation and immune regulation (Gao et al. 2023). Our study, through scRNA-seq and transcriptomic analysis, revealed high expression of CAPRIN1 in TNBC and experimentally confirmed the targeted regulation of CAPRIN1 by miR-497-5p. This discovery not only enhances our understanding of CAPRIN1’s function but also identifies a new potential target for developing TNBC therapies. Similar research in other cancer types, such as hepatocellular carcinoma, has also emphasized the crucial role of RNA-binding proteins in cancer progression. Consistent with our hypothesis, RT-qPCR validation demonstrated distinct expression patterns between parental and resistant TNBC cells. Specifically, LINC02544 and CAPRIN1 were significantly upregulated, whereas miR-497-5p was markedly downregulated in resistant MDA-MB-231/PEM cells compared to parental MDA-MB-231 cells. These findings further support the regulatory role of the LINC02544/miR-497-5p/CAPRIN1 axis in mediating immunotherapy resistance, reinforcing the notion that dysregulation of this pathway contributes to altered cellular behavior and resistance phenotypes.

Exos, crucial mediators of intercellular communication, play significant roles in cancer development, progression, and treatment (Bortoluzzi et al. 2017). Previous research has demonstrated that exos can carry various biomolecules, including RNA, proteins, and lipids, thus modulating the function of recipient cells (Skryabin et al. 2022). In this study, we utilized electroporation to load si-LINCO2544 into exos and validated its efficacy in reversing resistance in both in vitro and in vivo models. Our findings highlight the potential of exos in delivering siRNA and enhancing immune therapy efficacy, offering new strategies for treating TNBC. Certainly, while liposomes are widely used as drug delivery systems in many studies, they face challenges in terms of immunogenicity and stability compared to exosomes (Shafiei et al. 2021). Exosomes exhibit lower immunogenicity and possess the ability to cross physiological barriers, such as the blood-brain barrier, making them promising candidates for therapeutic and diagnostic applications (Perocheau et al. 2021). Other studies have also explored the application of exos in drug delivery, underscoring their broad potential across multiple cancer treatment domains.

This study uniquely combines scRNA-seq, transcriptomics, and bioinformatics analysis to systematically reveal the mechanisms of the LINC02544/miR-497-5p/CAPRIN1 axis in TNBC immune therapy resistance and explores the potential of exo/si-LINC02544 in reversing this resistance. Unlike previous research, our study not only confirms the roles of LINC02544 and miR-497-5p in TNBC but also identifies the critical role of CAPRIN1 in this process for the first time. Additionally, using exo technology to deliver siRNA has increased the sensitivity of TNBC cells to immune therapy, providing new evidence for the application of exos in cancer treatment. This aligns with our research strategies in other cancer types, emphasizing the importance of a multi-layered, comprehensive research approach.

Our study demonstrates that LINC02544 mediates immune therapy resistance in TNBC via the miR-497-5p/CAPRIN1 axis and confirms the potential of exo/si-LINC02544 in reversing this resistance. The results show that exo/si-LINC02544 significantly reduce the expression of LINC02544 and CAPRIN1 in resistant TNBC cells, increase miR-497-5p expression, and inhibit cell proliferation and migration. Moreover, the combination of exo/si-LINC02544 with PD-1 inhibitors significantly suppressed tumor growth in mice and enhanced immune cell infiltration. These findings not only provide new potential targets and therapeutic strategies for treating TNBC immune resistance but also offer important scientific evidence and clinical guidance for the application of exos in cancer therapy.

However, this study has limitations, including a small sample size and experimental constraints. Future research should expand the sample size and explore the role of the LINC02544/miR-497-5p/CAPRIN1 axis in other cancer types. Moreover, we plan to explore more mature drug delivery systems, such as lipid nanoparticles (LNPs), and further optimize the exosome-based delivery system to enhance its efficacy and safety in clinical applications.

## Conclusion

**Fig. 10:**
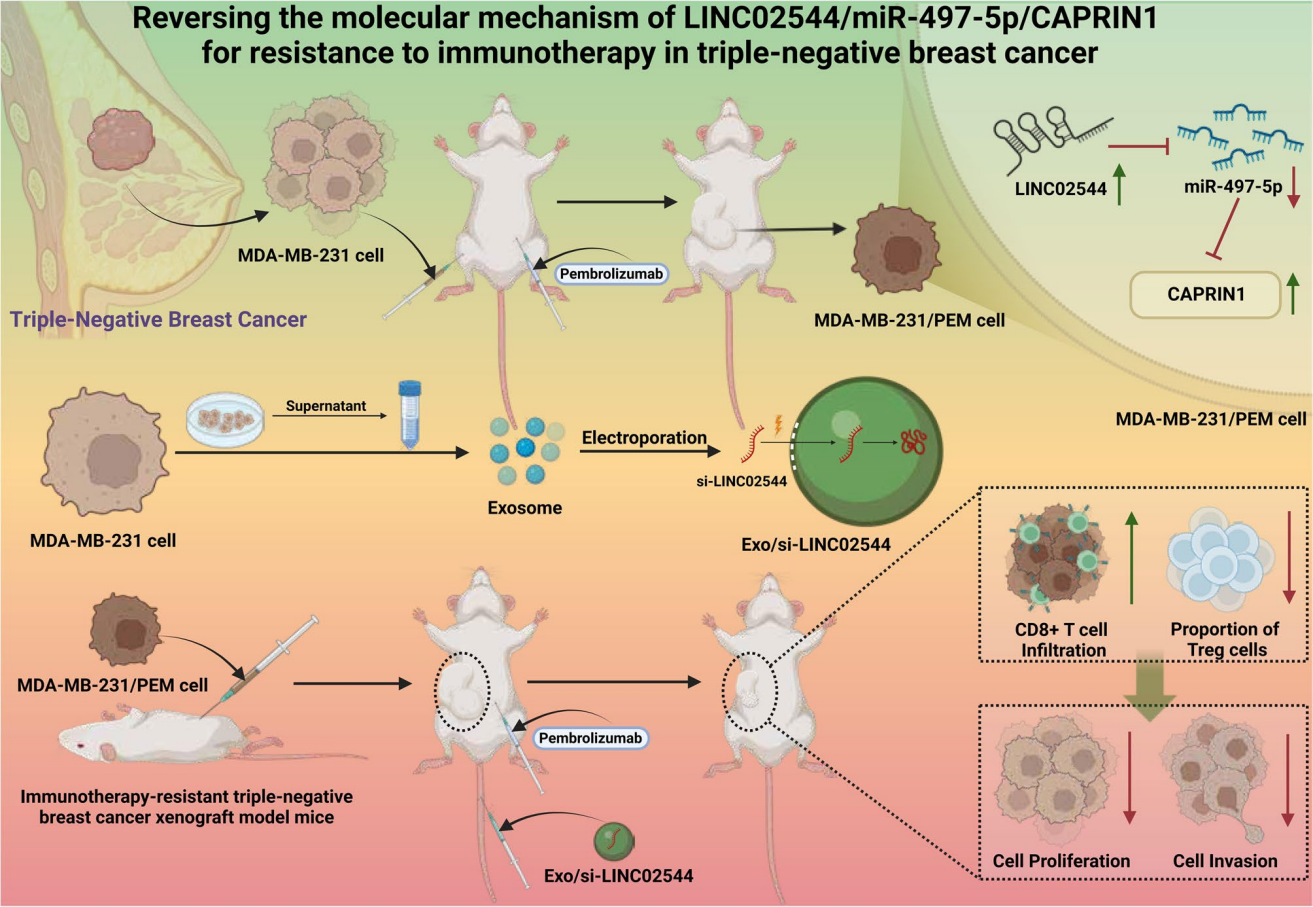
Molecular Mechanism of LINC02544/miR-497-5p/CAPRIN1 in Reversing Immune Therapy Resistance in TNBC

This study systematically reveals the mechanism by which LINC02544 promotes immune therapy resistance in TNBC by inhibiting miR-497-5p and upregulating CAPRIN1 (Mechanism Diagram). Using CRISPR/Cas9 and siRNA technology to knock down LINC02544 significantly inhibited CAPRIN1 expression and activated miR-497-5p. Additionally, exo/si-LINC02544 effectively suppressed the proliferation and migration of resistant TNBC cells. In vivo experiments demonstrated that combining exo/si-LINC02544 with PD-L1 inhibitors significantly inhibited tumor growth, increased CD8 + T cell infiltration, and reduced the proportion of Treg cells (Fig. 10), suggesting potential in reversing immune therapy resistance.

This study is the first to highlight the core role of the LINC02544/miR-497-5p/CAPRIN1 axis in TNBC immune therapy resistance, providing a novel molecular target. The strategy of exo/si-LINC02544 not only shows significant efficacy in vitro and in vivo but also demonstrates potential in reducing immune therapy resistance in clinical applications. This approach could offer new treatment options for TNBC patients, enhancing the effectiveness of immune therapy and improving patient outcomes. However, the study has limitations, including the inability to fully replicate the clinical environment, and the delivery efficiency and stability of exo-loaded siRNA require further optimization. Future research should explore the role of LINC02544 in other cancer types to validate its feasibility as a broad therapeutic target. Looking ahead, with technological advancements and more clinical trials, exo-loaded siRNA holds promise as a safe and effective precision therapy, advancing the development of personalized treatment strategies.

## Supplementary Information

Supplementary Material 1.

Supplementary Material 2.

Supplementary Material 3.

Supplementary Material 4.

Supplementary Material 5.

Supplementary Material 6.

Supplementary Material 7.

## Data Availability

The datasets generated and/or analyzed during the current study are not publicly available due to privacy and confidentiality agreements with the participants but are available from the corresponding author on reasonable request.

## Abbreviations

ANOVA: Analysis of Variance
ATCC: American Type Culture Collection
BRCA: Breast Invasive Carcinoma
CCK-8: Cell Counting Kit-8
CNVs: Copy Number Variations
CSB: Cell Staining Buffer
DEGs: Differentially Expressed Genes
ER: Estrogen Receptors
Exo: Exosome
Exo/si-LINC02544: Exosome-loaded LINC02544 siRNA
GEO: Gene Expression Omnibus
HCC: Hepatocellular Carcinoma
H&E: Hematoxylin and Eosin
HER2: Human Epidermal Growth Factor Receptor 2
HSD: Honestly Significant Difference
IHC: Immunohistochemistry
LASSO: Least Absolute Shrinkage and Selection Operator
LncRNA: Long Non-coding RNA
MAD: Median Absolute Deviation
MDSCs: Myeloid-derived Suppressor Cells
MiRNA: microRNA
NTA: Nanoparticle Tracking Analysis
PCA: Principal Component Analysis
PCs: Principal Components
PI: Propidium Iodide
qRT-PCR: Quantitative Reverse Transcription Polymerase Chain Reaction
ROC: Receiver Operating Characteristic
scRNA-seq: Single-cell Sequencing
SsGSEA: Single-Sample Gene Set Enrichment Analysis
TEM: Transmission Electron Microscopy
TCGA: The Cancer Genome Atlas
TILs: Tumor-infiltrating Lymphocytes
Tregs: Regulatory T Cells
TNBC: Triple-negative Breast Cancer
WB: Western Blot
WT: Wild-type
WGCNA: Weighted Gene Co-expression Network Analysis
