# Supplementary figures and images for: Targeting LINC02544/miR-497-5p/CAPRIN1 axis via exosome-based siRNA to overcome immunotherapy resistance in triple-negative breast cancer
Source: Mol Med. 2025 Aug 16;31:278. doi: 10.1186/s10020-025-01336-w (PMC12357379; doi:10.1186/s10020-025-01336-w)

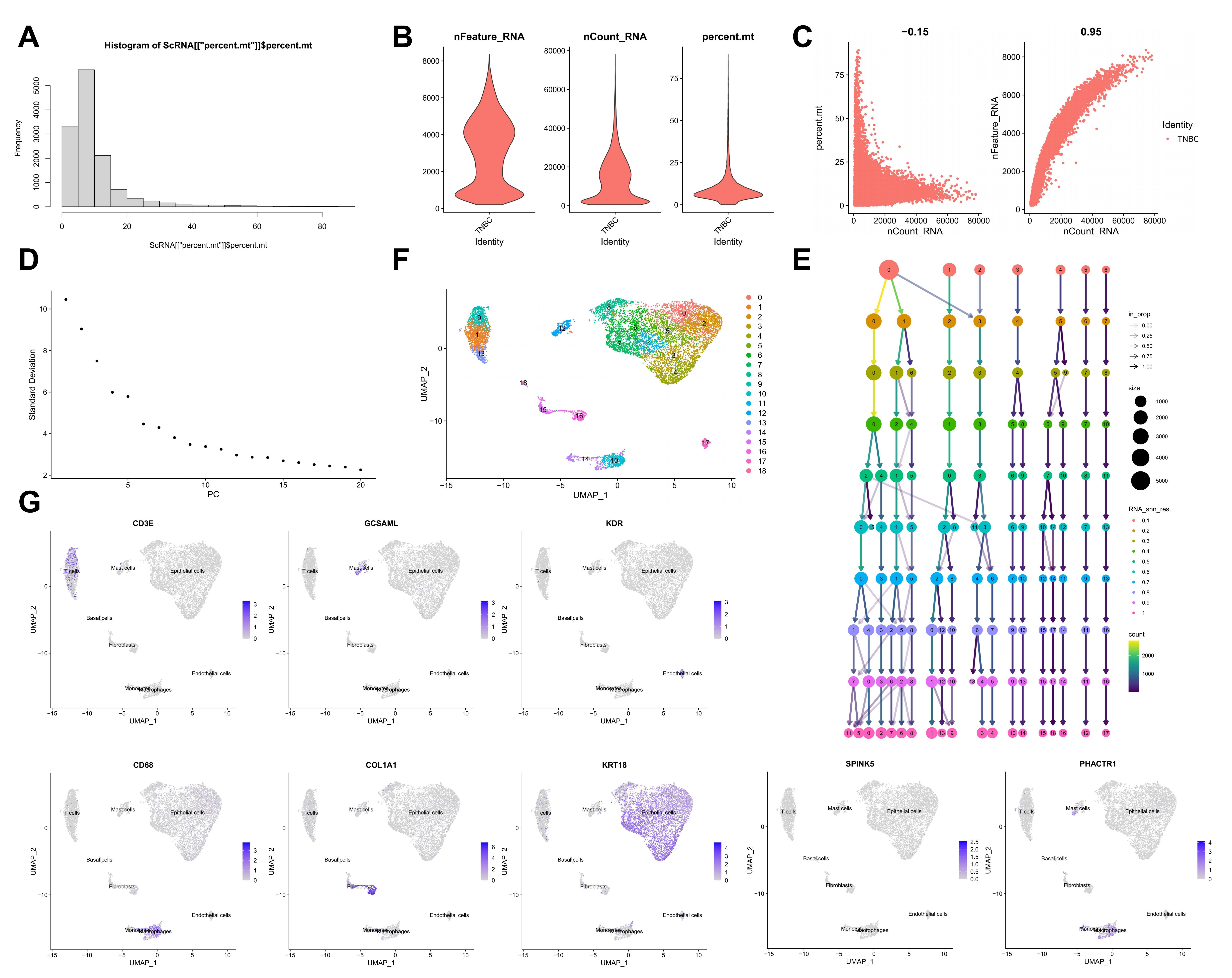

Supplement: Supplementary file 1 — Supplementary Material 1. [file 10020_2025_1336_MOESM1_ESM.jpg]

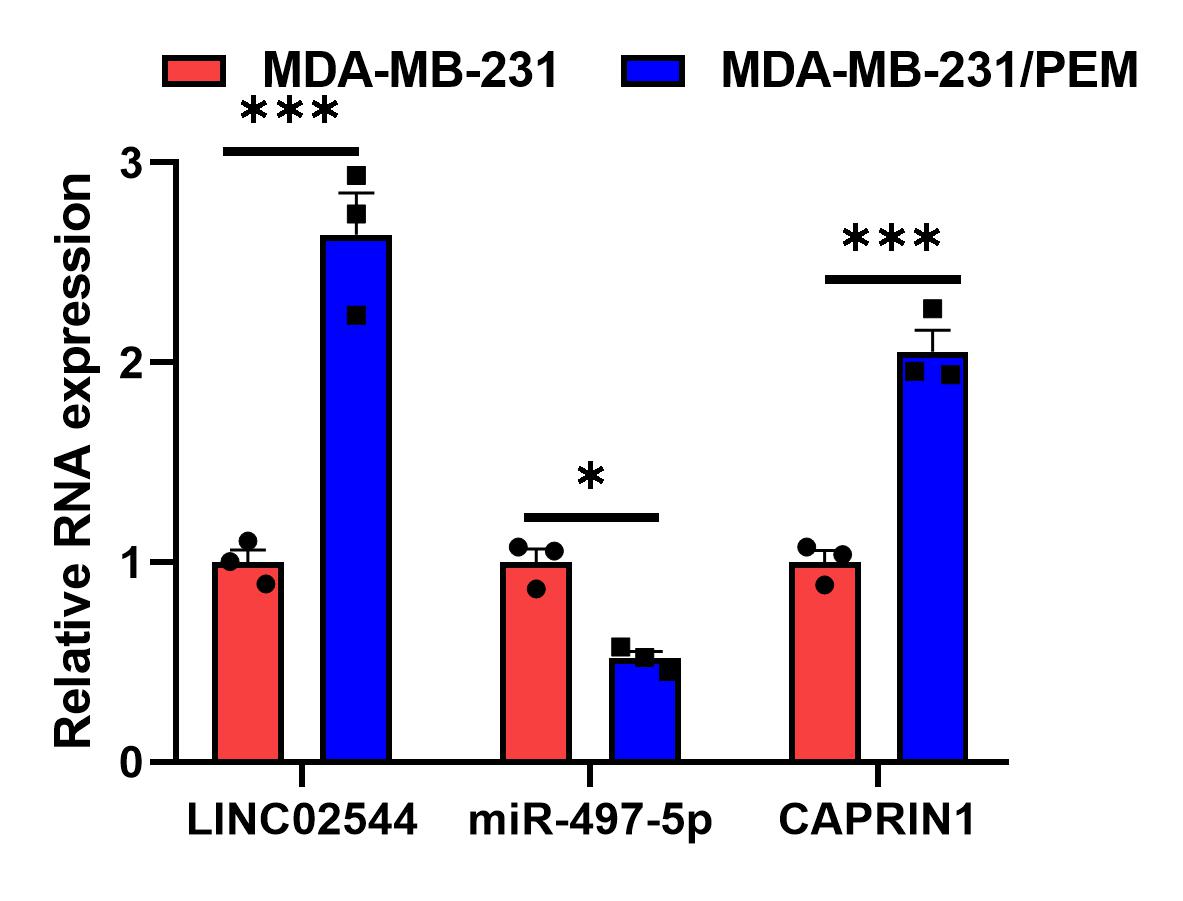

Supplement: Supplementary file 2 — Supplementary Material 2. [file 10020_2025_1336_MOESM2_ESM.jpg]

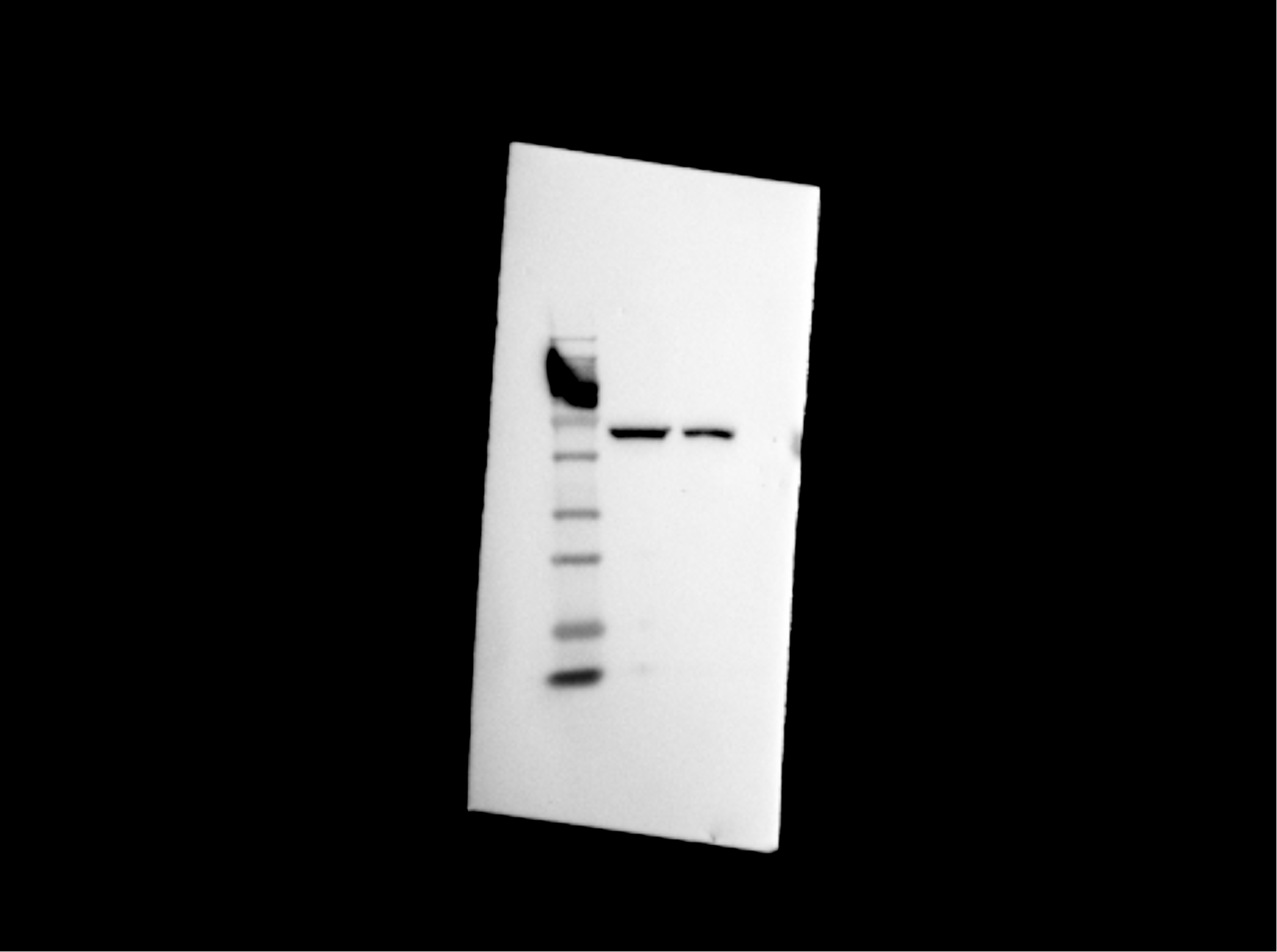

Supplement: Supplementary file 4 — Supplementary Material 4. [file 10020_2025_1336_MOESM4_ESM.zip › full uncropped Gels and Blots image(s) of figure 6/full uncropped Gels and Blots image(s) of figure 6F-1.jpg]

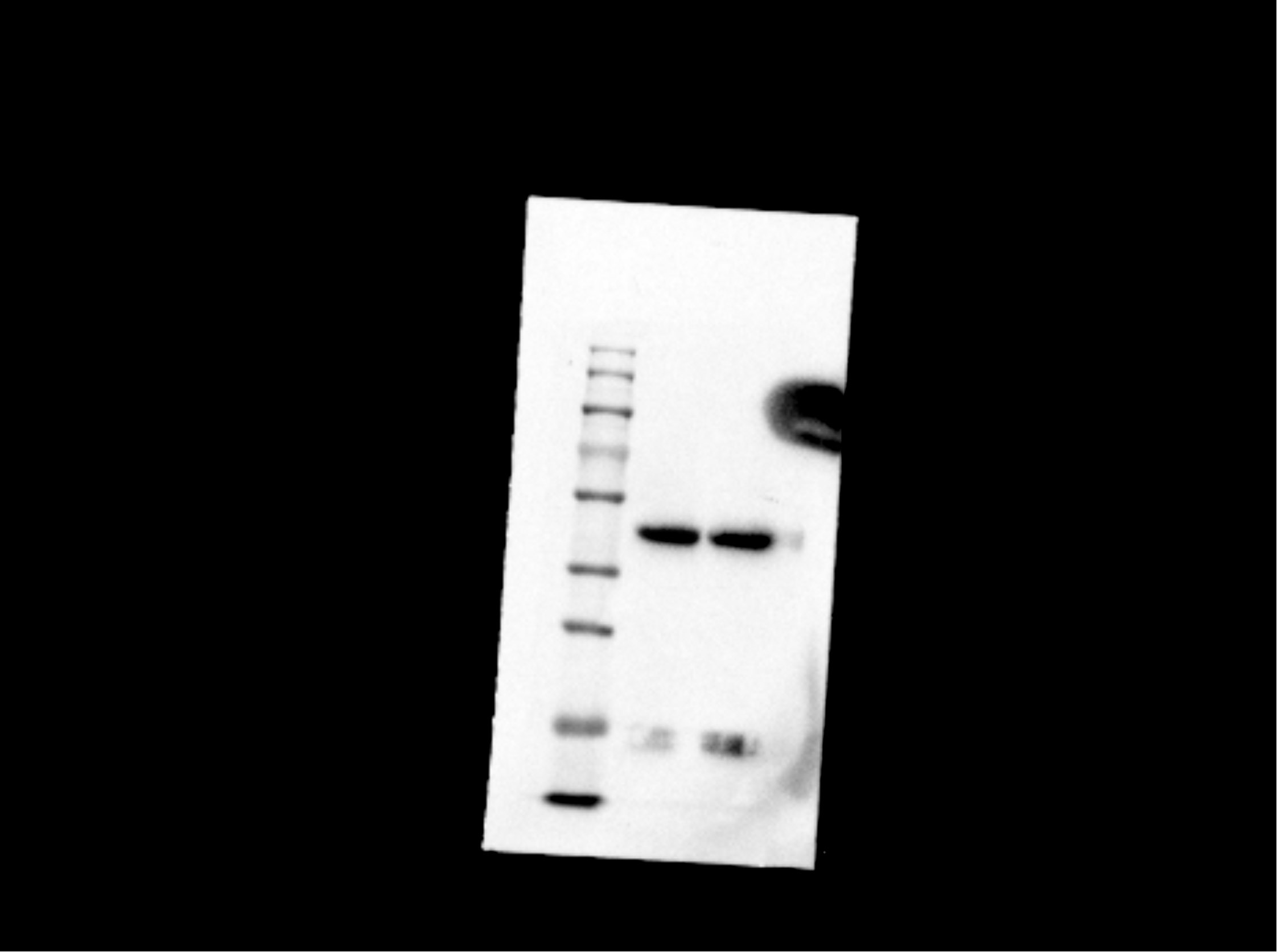

Supplement: Supplementary file 4 — Supplementary Material 4. [file 10020_2025_1336_MOESM4_ESM.zip › full uncropped Gels and Blots image(s) of figure 6/full uncropped Gels and Blots image(s) of figure 6F-2.jpg]

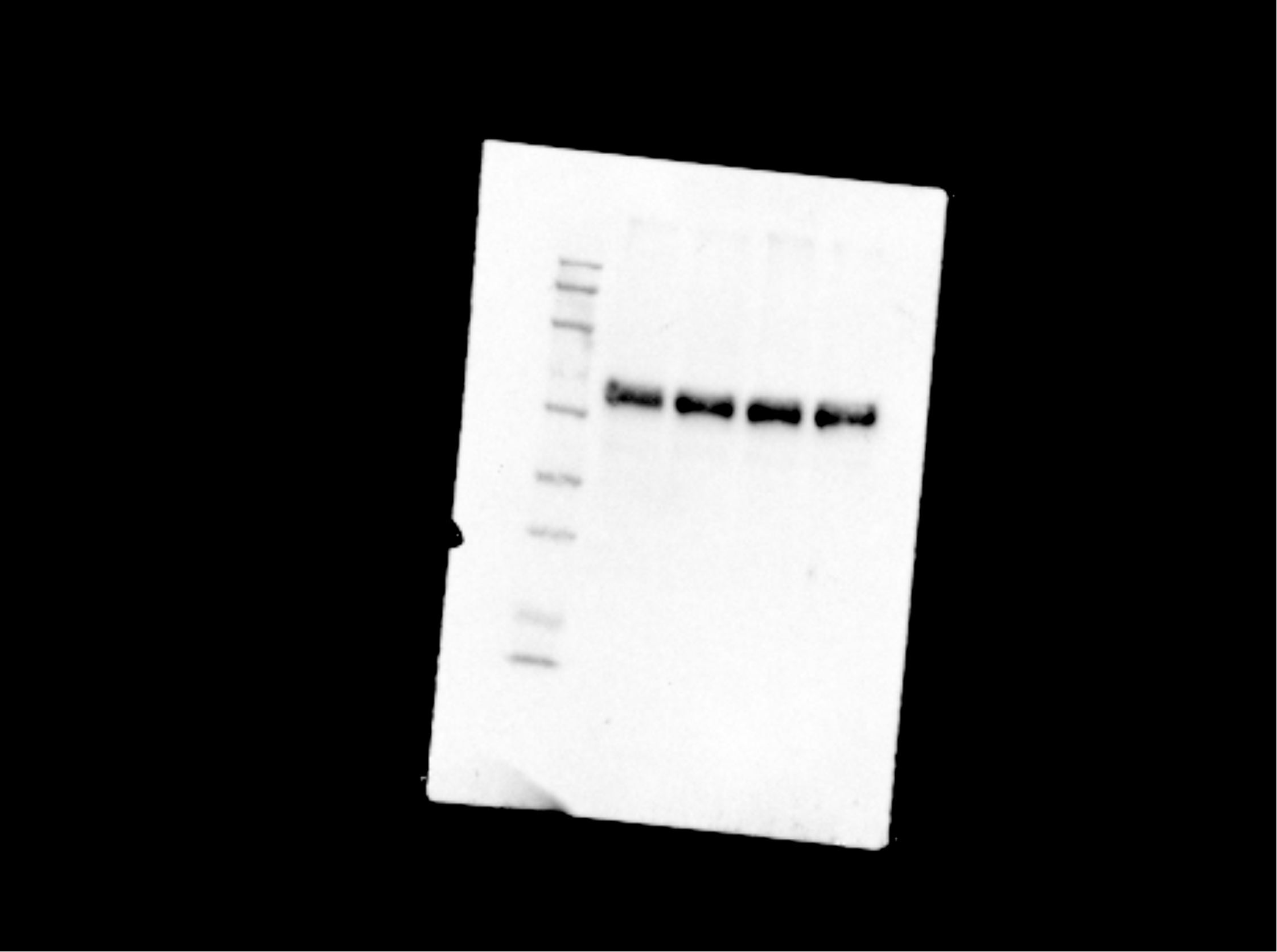

Supplement: Supplementary file 4 — Supplementary Material 4. [file 10020_2025_1336_MOESM4_ESM.zip › full uncropped Gels and Blots image(s) of figure 6/full uncropped Gels and Blots image(s) of figure 6H-1.jpg]

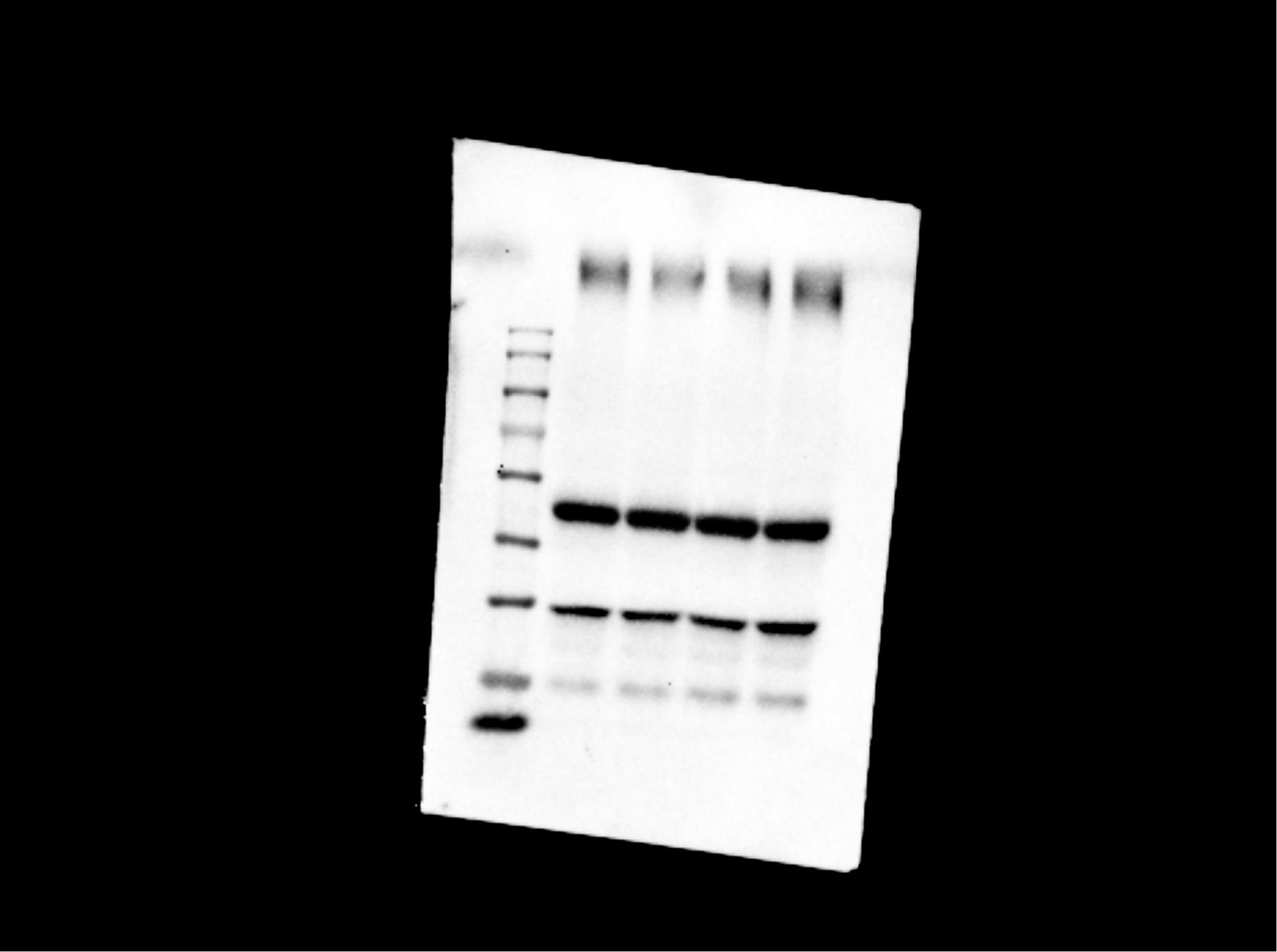

Supplement: Supplementary file 4 — Supplementary Material 4. [file 10020_2025_1336_MOESM4_ESM.zip › full uncropped Gels and Blots image(s) of figure 6/full uncropped Gels and Blots image(s) of figure 6H-2.jpg]

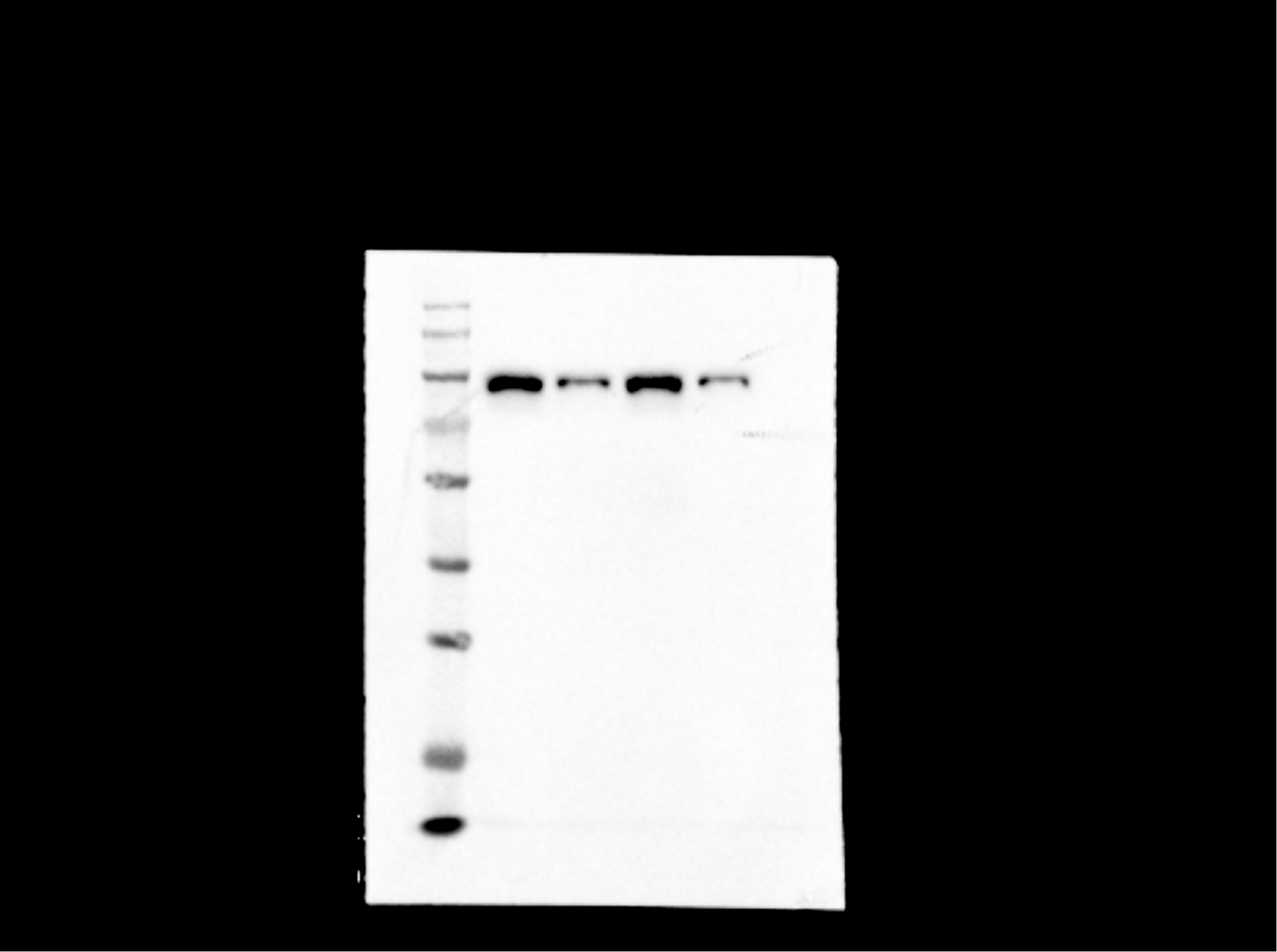

Supplement: Supplementary file 5 — Supplementary Material 5. [file 10020_2025_1336_MOESM5_ESM.zip › full uncropped Gels and Blots image(s) of figure 9/full uncropped Gels and Blots image(s) of figure 9E-1.jpg]

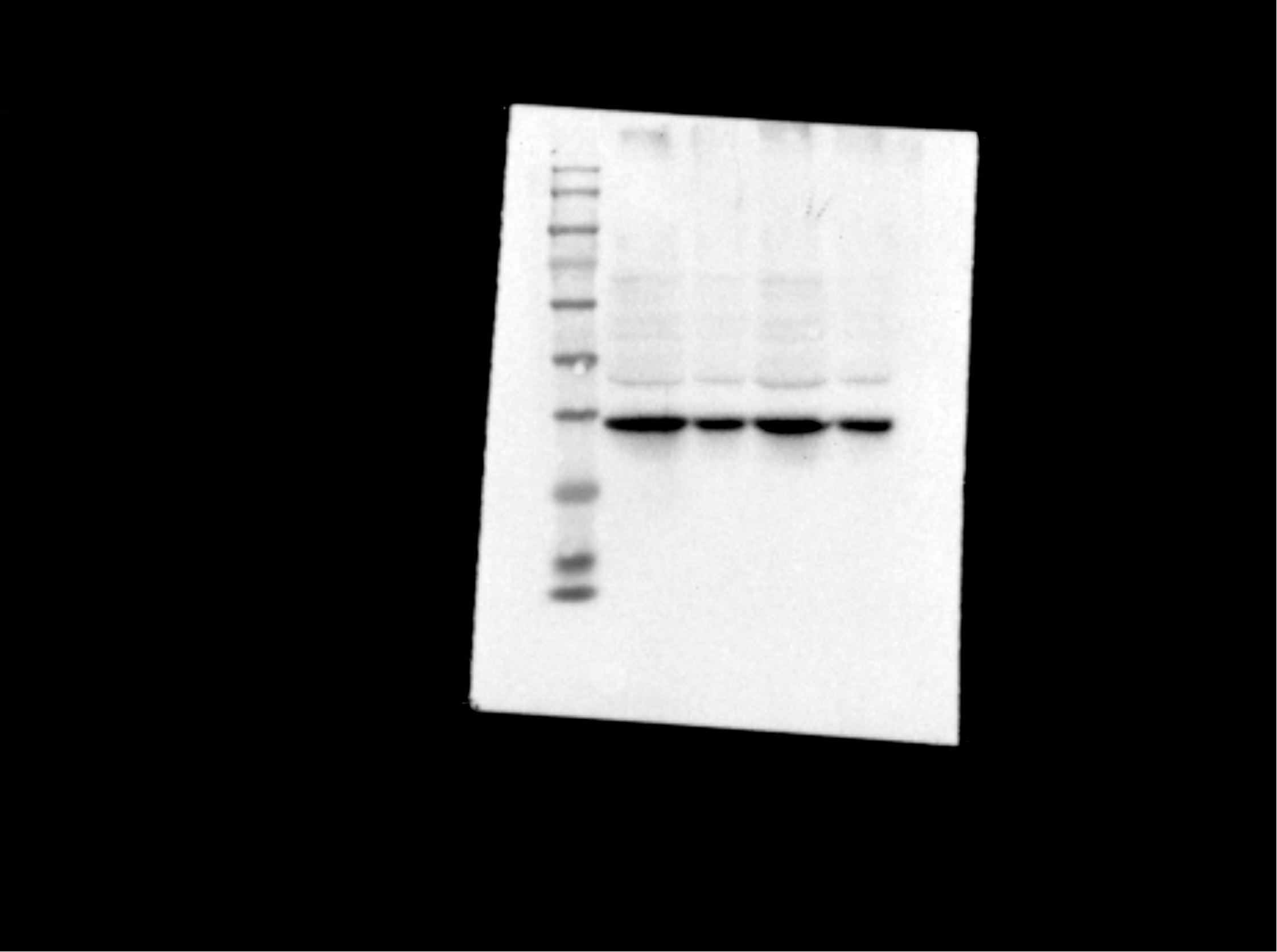

Supplement: Supplementary file 5 — Supplementary Material 5. [file 10020_2025_1336_MOESM5_ESM.zip › full uncropped Gels and Blots image(s) of figure 9/full uncropped Gels and Blots image(s) of figure 9E-2.jpg]

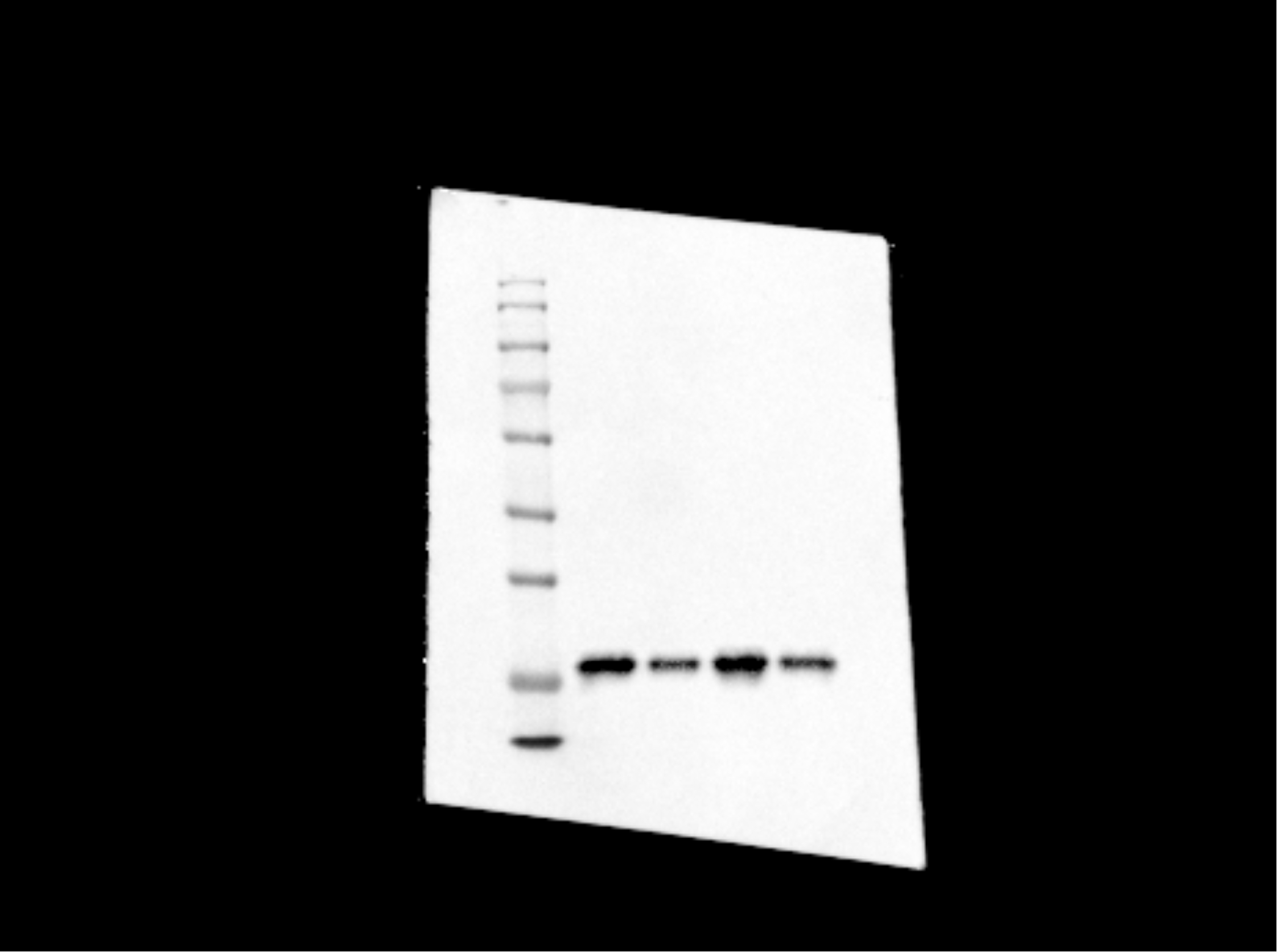

Supplement: Supplementary file 5 — Supplementary Material 5. [file 10020_2025_1336_MOESM5_ESM.zip › full uncropped Gels and Blots image(s) of figure 9/full uncropped Gels and Blots image(s) of figure 9E-3.jpg]

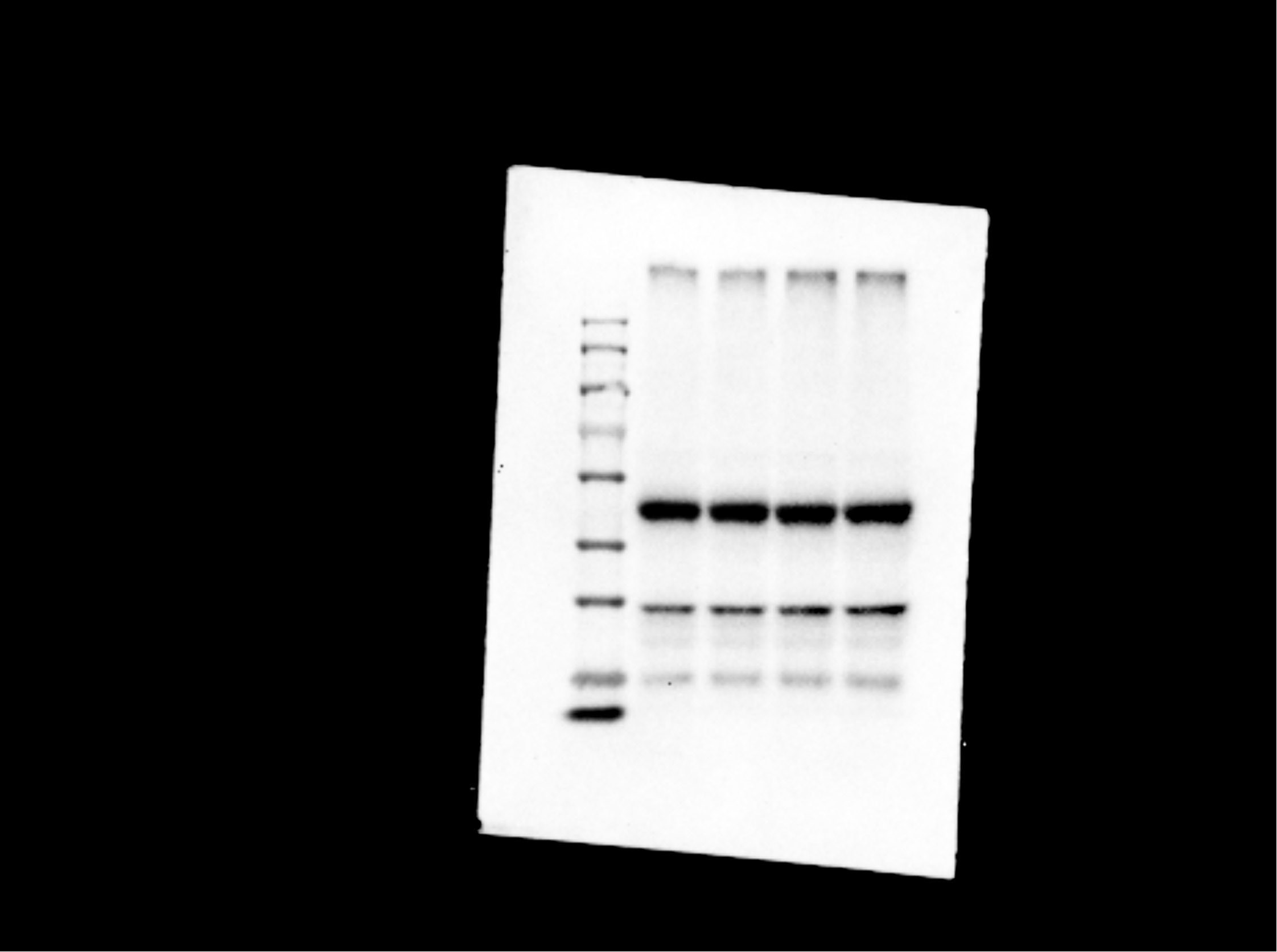

Supplement: Supplementary file 5 — Supplementary Material 5. [file 10020_2025_1336_MOESM5_ESM.zip › full uncropped Gels and Blots image(s) of figure 9/full uncropped Gels and Blots image(s) of figure 9E-4.jpg]

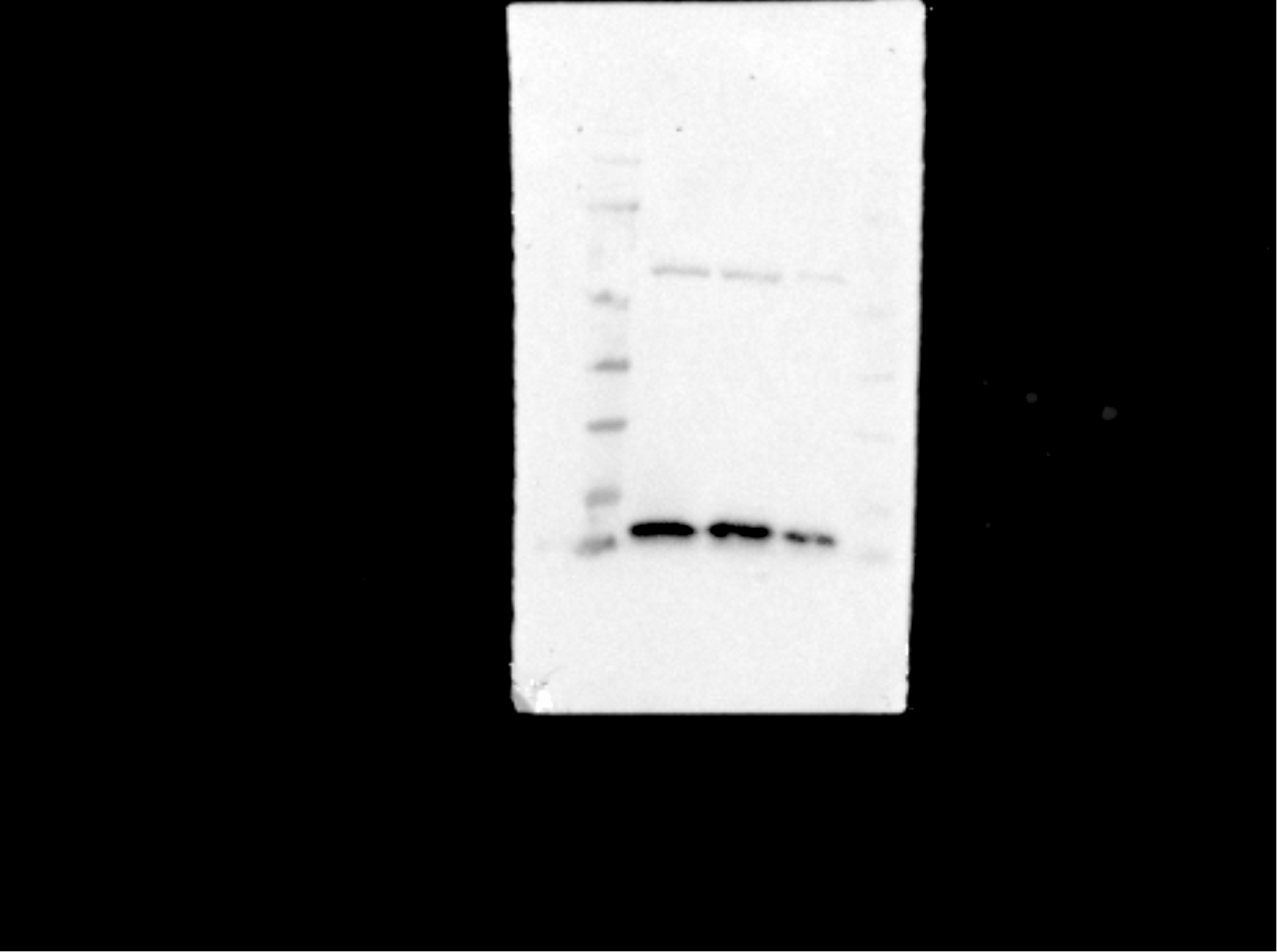

Supplement: Supplementary file 6 — Supplementary Material 6. [file 10020_2025_1336_MOESM6_ESM.zip › full uncropped Gels and Blots image(s) of figure 7/full uncropped Gels and Blots image(s) of figure 7B-1.jpg]

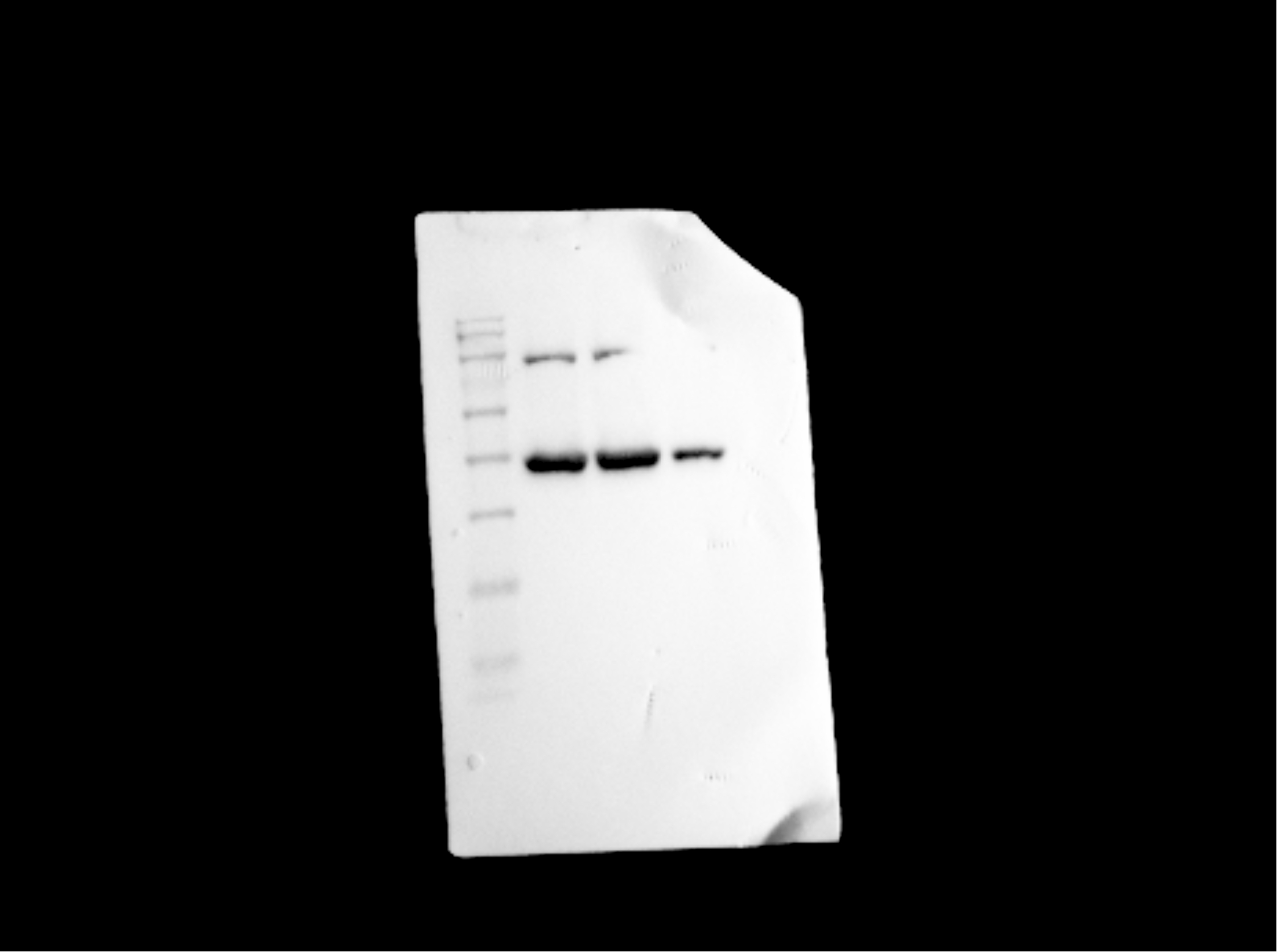

Supplement: Supplementary file 6 — Supplementary Material 6. [file 10020_2025_1336_MOESM6_ESM.zip › full uncropped Gels and Blots image(s) of figure 7/full uncropped Gels and Blots image(s) of figure 7B-2.jpg]

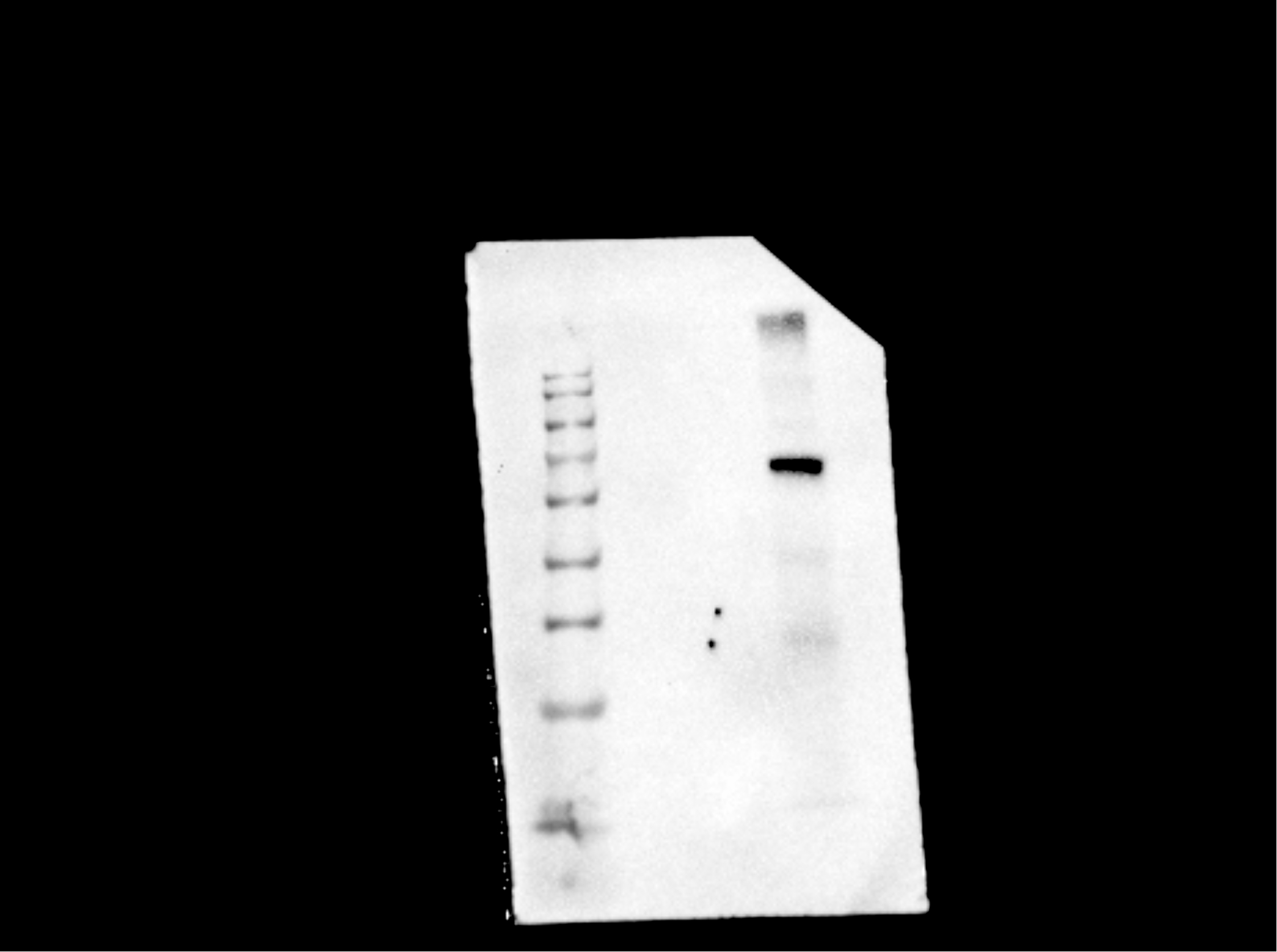

Supplement: Supplementary file 6 — Supplementary Material 6. [file 10020_2025_1336_MOESM6_ESM.zip › full uncropped Gels and Blots image(s) of figure 7/full uncropped Gels and Blots image(s) of figure 7B-3.jpg]

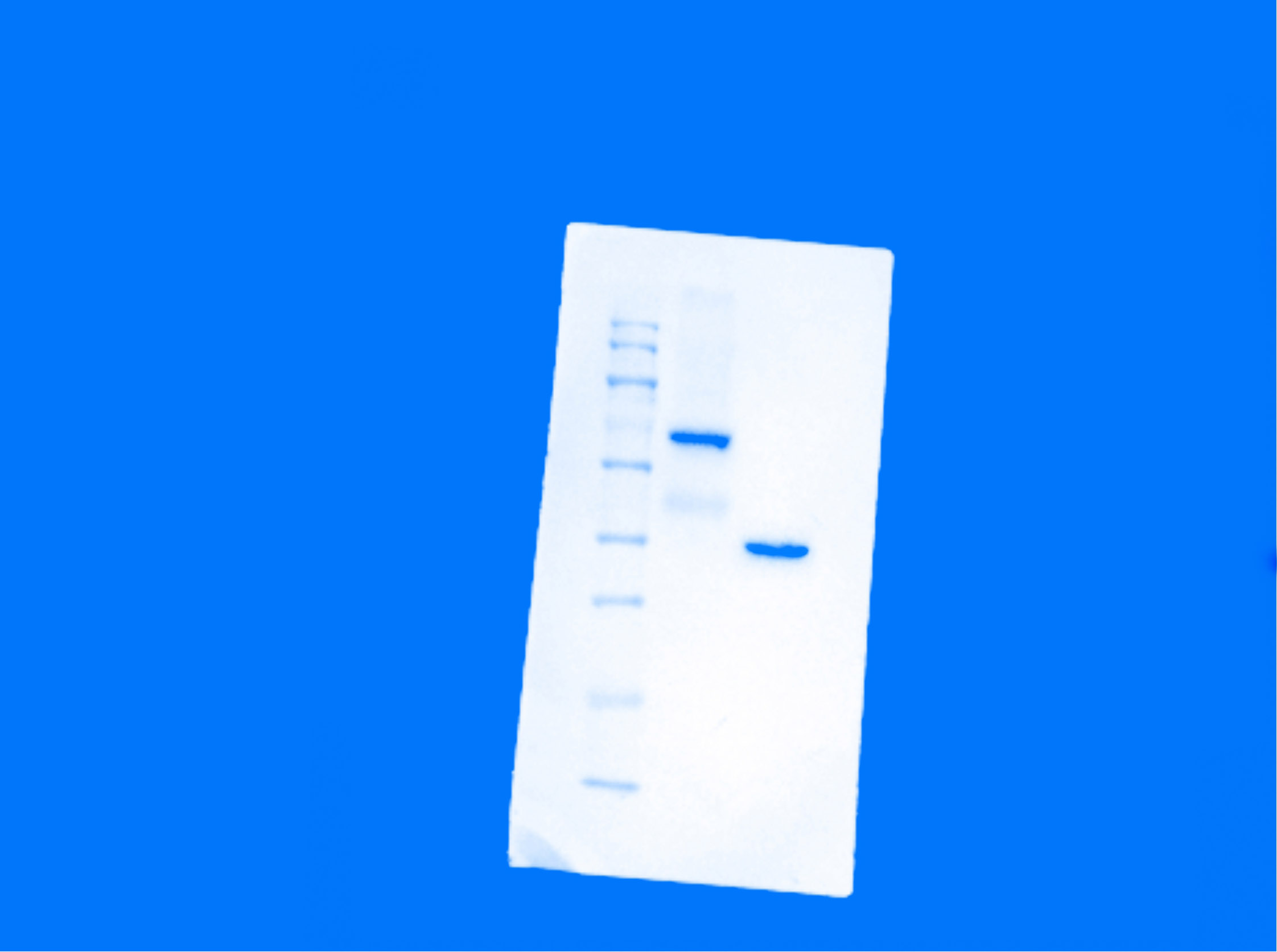

Supplement: Supplementary file 6 — Supplementary Material 6. [file 10020_2025_1336_MOESM6_ESM.zip › full uncropped Gels and Blots image(s) of figure 7/full uncropped Gels and Blots image(s) of figure 7C-1.jpg]

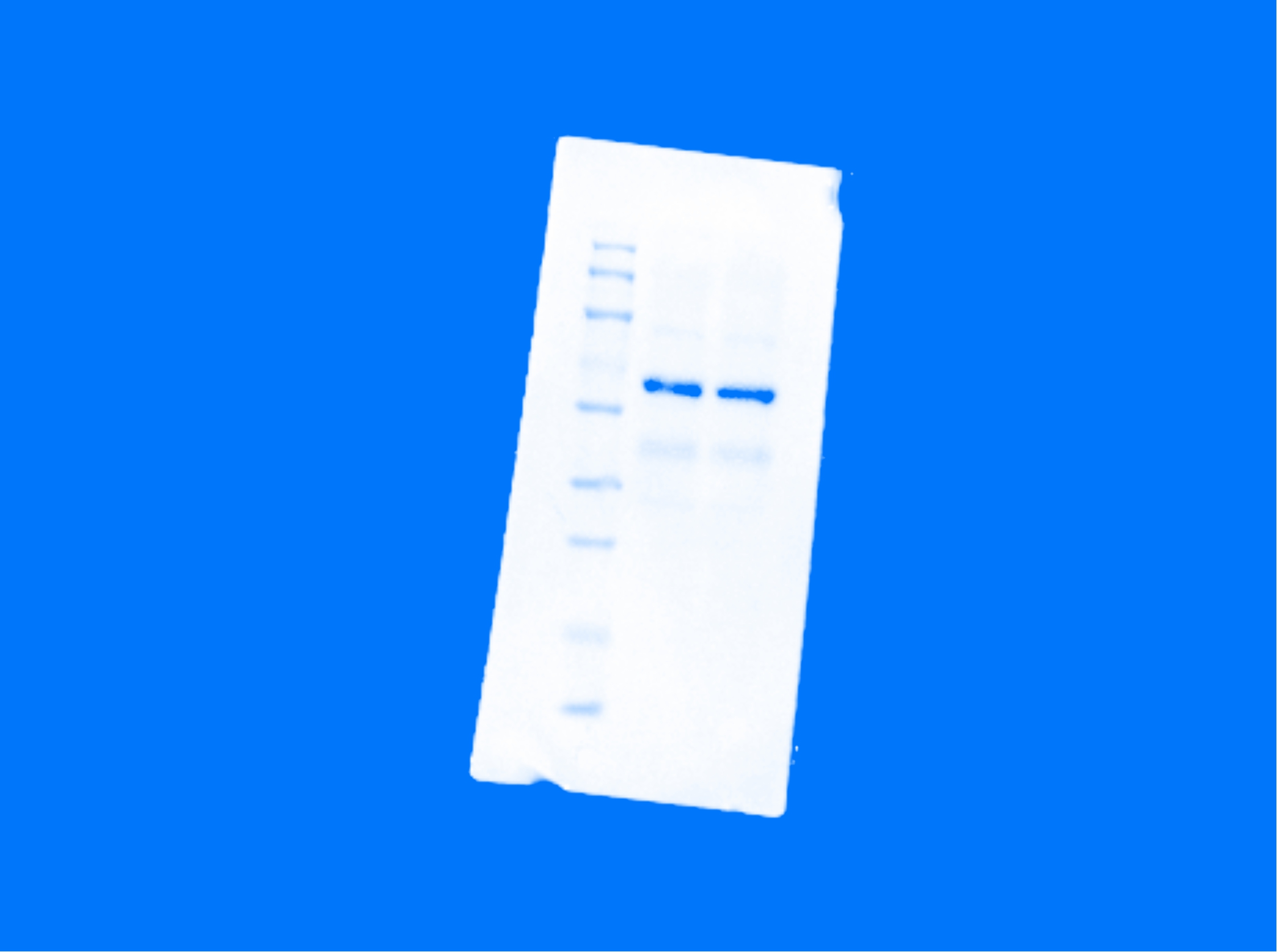

Supplement: Supplementary file 6 — Supplementary Material 6. [file 10020_2025_1336_MOESM6_ESM.zip › full uncropped Gels and Blots image(s) of figure 7/full uncropped Gels and Blots image(s) of figure 7C-2.jpg]

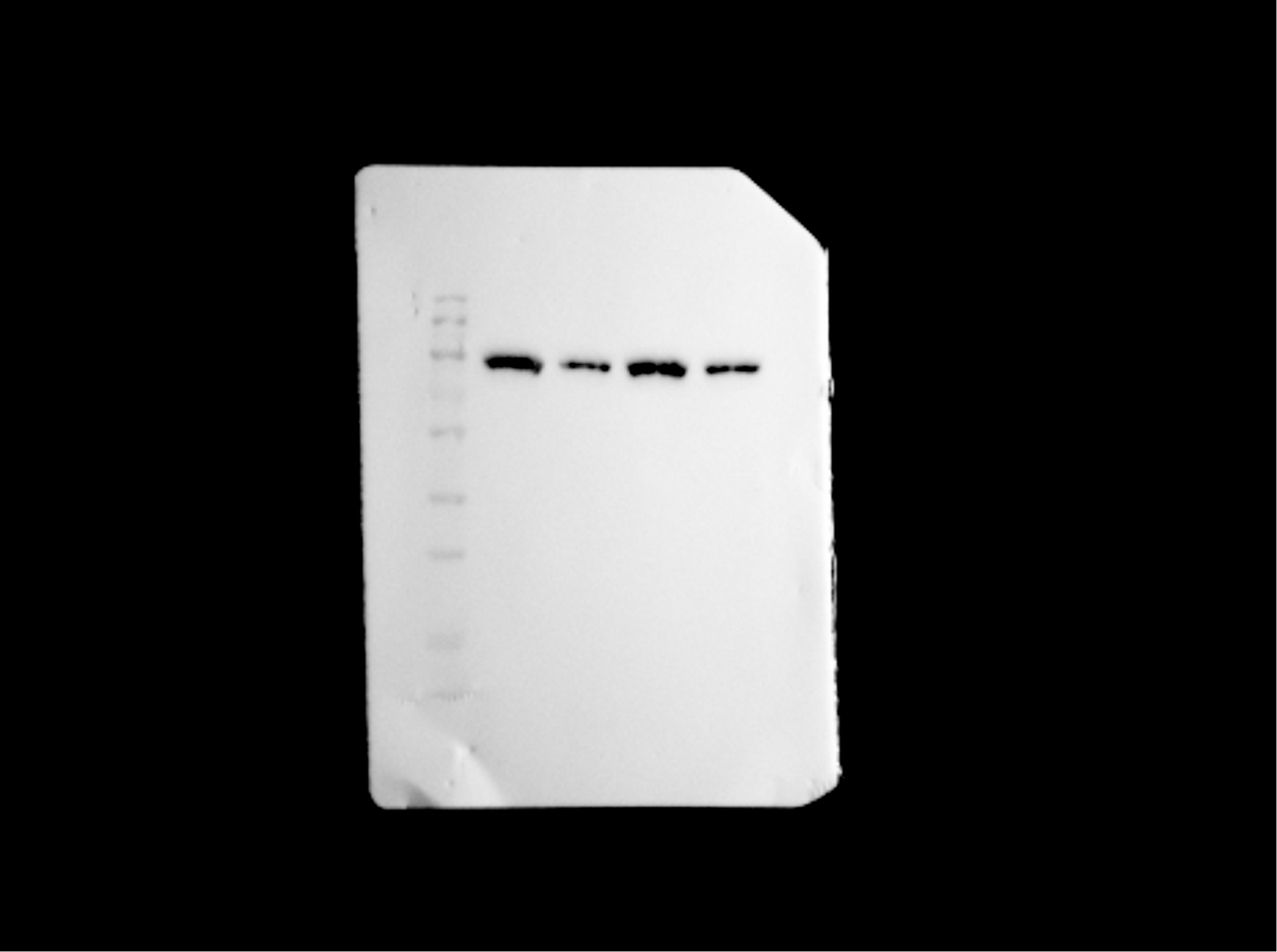

Supplement: Supplementary file 7 — Supplementary Material 7. [file 10020_2025_1336_MOESM7_ESM.zip › full uncropped Gels and Blots image(s) of figure 8/full uncropped Gels and Blots image(s) of figure 8B-1.jpg]

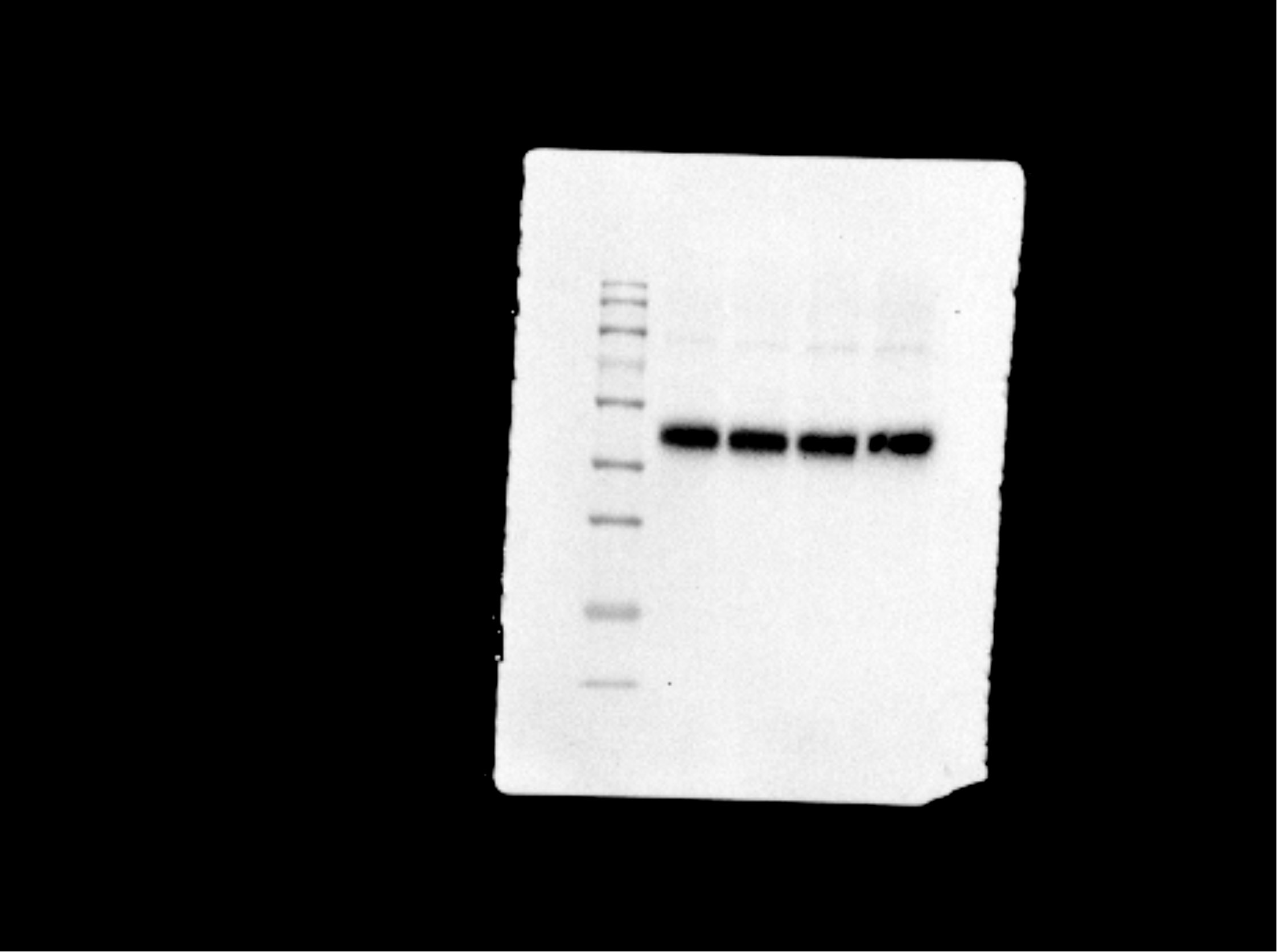

Supplement: Supplementary file 7 — Supplementary Material 7. [file 10020_2025_1336_MOESM7_ESM.zip › full uncropped Gels and Blots image(s) of figure 8/full uncropped Gels and Blots image(s) of figure 8B-2.jpg]

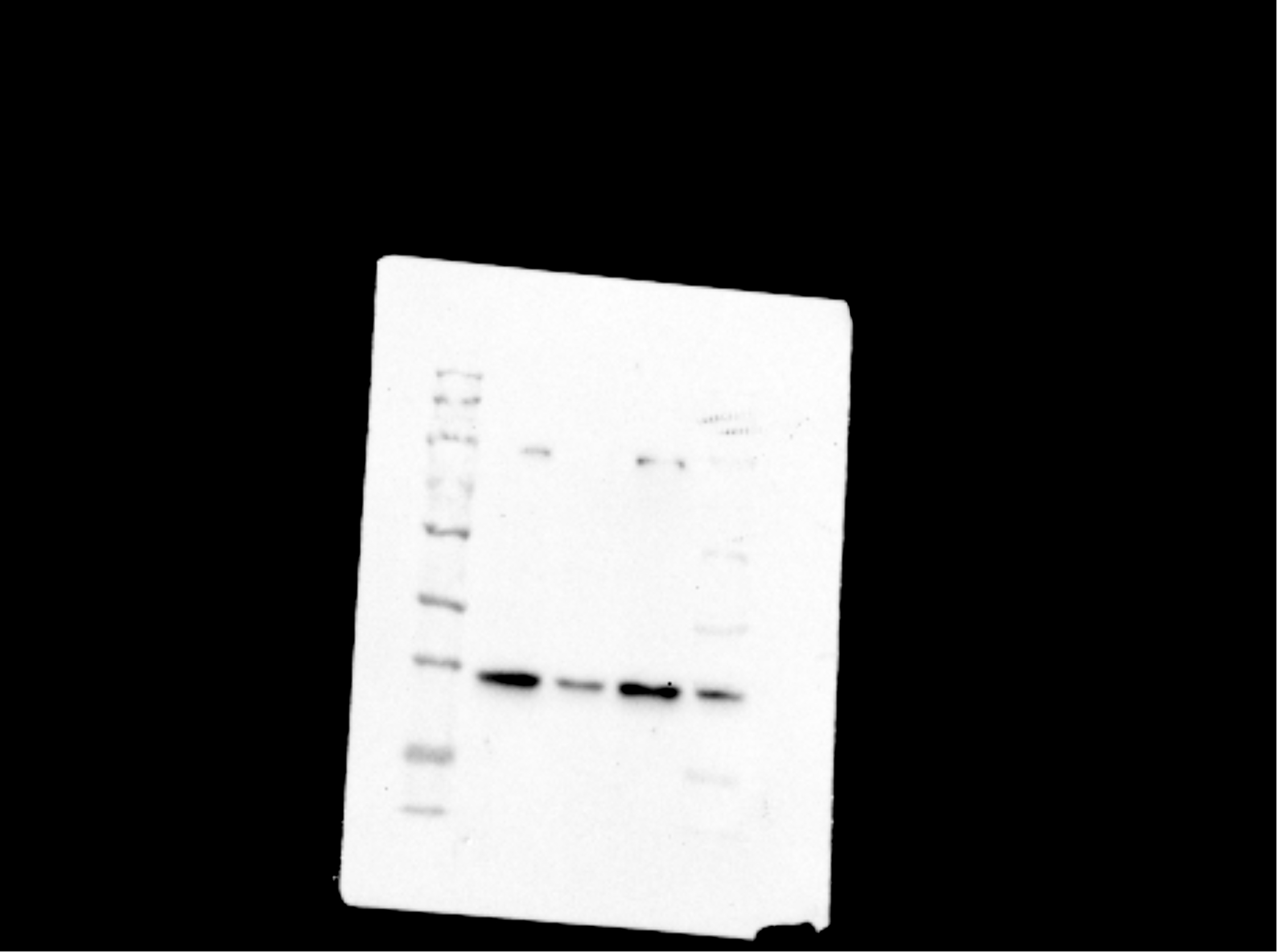

Supplement: Supplementary file 7 — Supplementary Material 7. [file 10020_2025_1336_MOESM7_ESM.zip › full uncropped Gels and Blots image(s) of figure 8/full uncropped Gels and Blots image(s) of figure 8H-1.jpg]

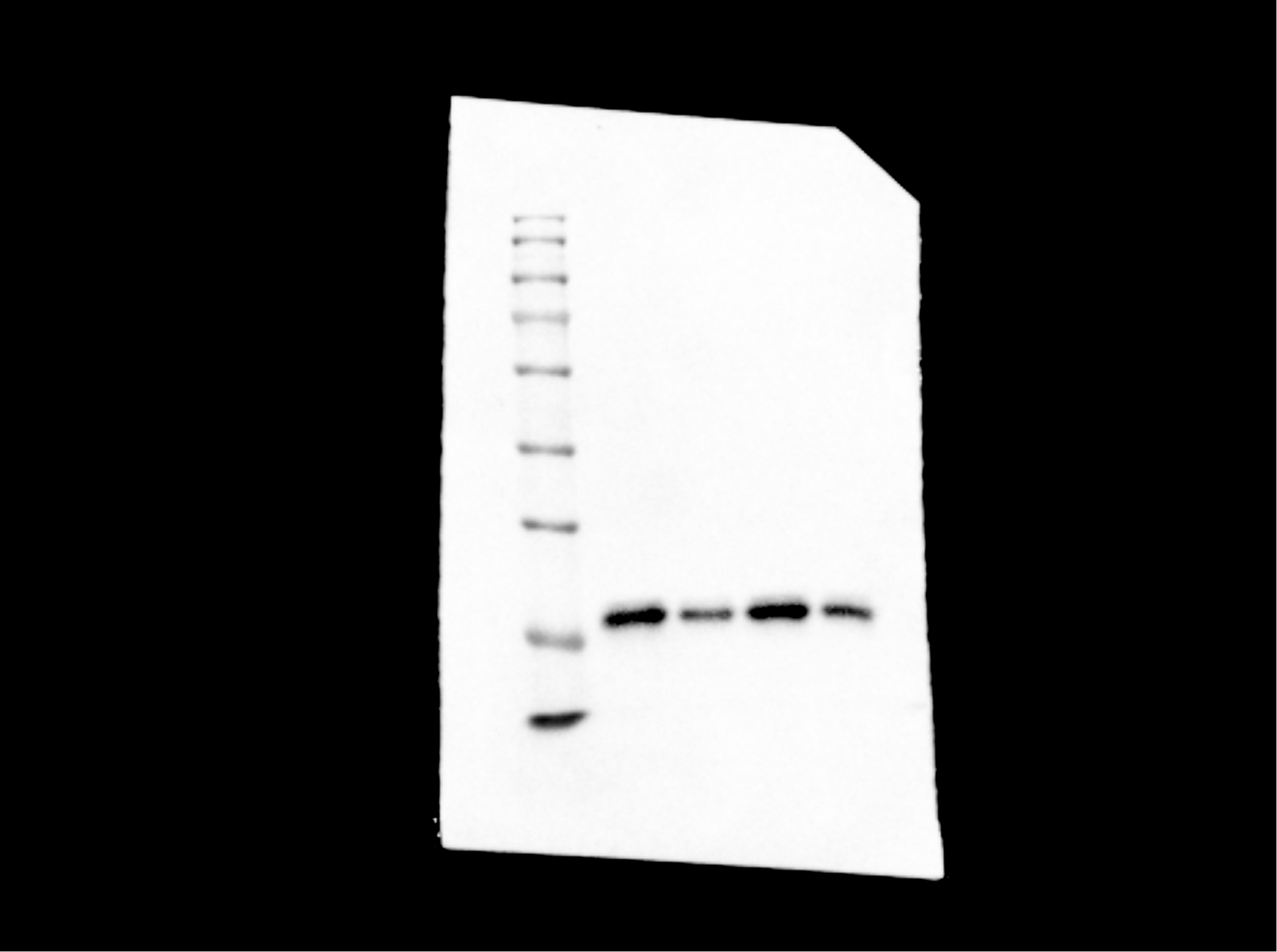

Supplement: Supplementary file 7 — Supplementary Material 7. [file 10020_2025_1336_MOESM7_ESM.zip › full uncropped Gels and Blots image(s) of figure 8/full uncropped Gels and Blots image(s) of figure 8H-2.jpg]

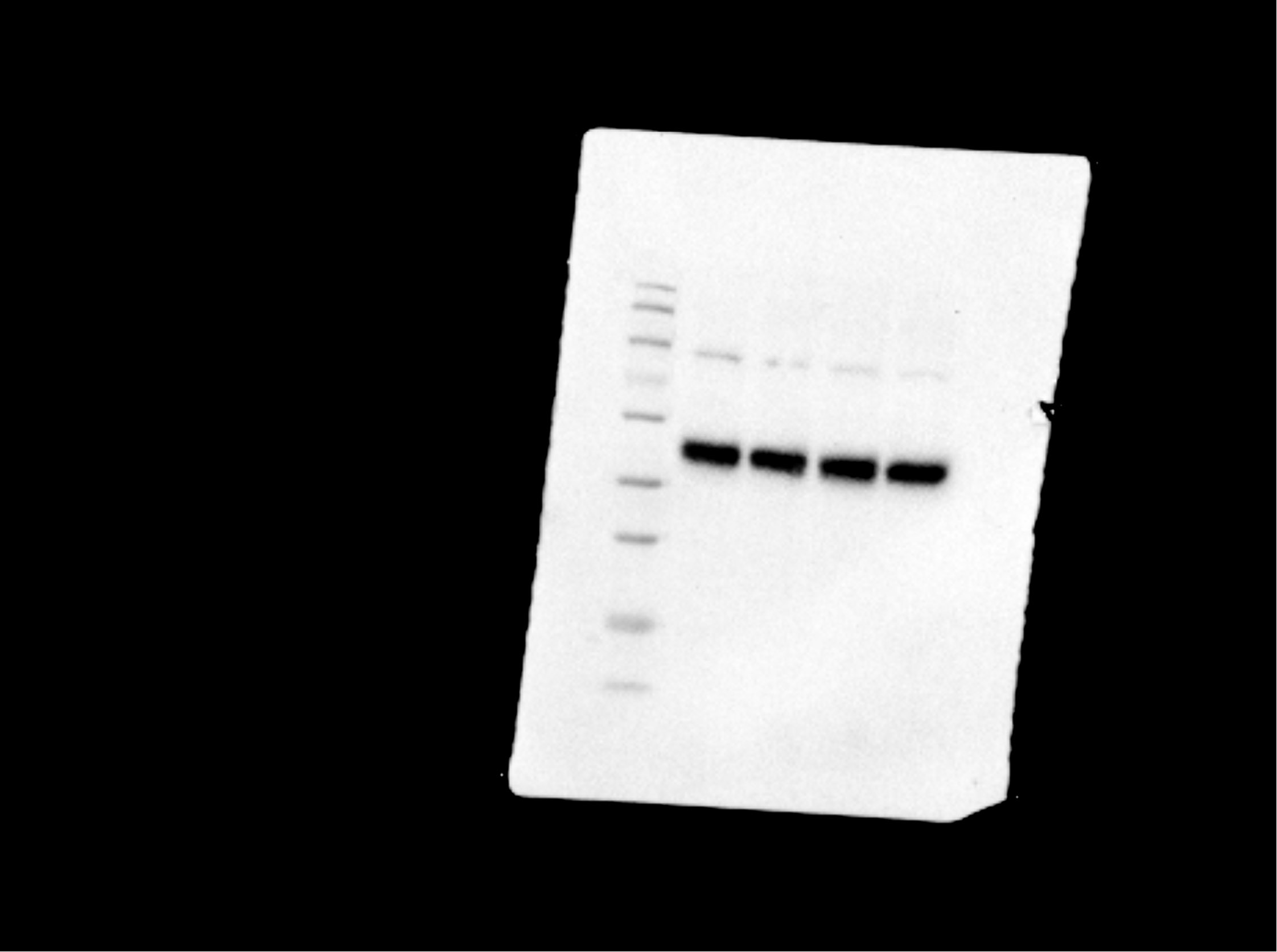

Supplement: Supplementary file 7 — Supplementary Material 7. [file 10020_2025_1336_MOESM7_ESM.zip › full uncropped Gels and Blots image(s) of figure 8/full uncropped Gels and Blots image(s) of figure 8H-3.jpg]
