# Supplementary material for: Targeting LINC02544/miR-497-5p/CAPRIN1 axis via exosome-based siRNA to overcome immunotherapy resistance in triple-negative breast cancer
Source: Mol Med. 2025 Aug 16;31:278. doi: 10.1186/s10020-025-01336-w (PMC12357379; doi:10.1186/s10020-025-01336-w)
Supplement: Supplementary file 3 — Supplementary Material 3. [file 10020_2025_1336_MOESM3_ESM.docx]

**Table S1. RT-qPCR primer sequence.**

| **Gene** | **Sequence(5'-3')** |
| --- | --- |
| miR-497-5p (human) | Forward: GCGCAGCAGCACACTGTG |
|  | Reverse: Reverse Universal Primer |
| CAPRIN1 (human) | Forward: GAAGTGCGGACTGACCTGAA |
|  | Reverse: CCACAGGTGAATGGAGGCAT |
| GAPDH (human) | Forward: GGCTGTTGTCATACTTCTCATGG |
|  | Reverse: GGAGCGAGATCCCTCCAAAAT |
| LINC02544 (human) | Forward: AAAGGACAACTCCTTGGGGC |
|  | Reverse: CAGTAGCTCTCACGCTCTCG |
| U6 (human) | Forward: CTCGCTTCGGCAGCACA |
|  | Reverse: Reverse Universal Primer |
